# Identification of skeletal muscle stem cell adhesion motifs using spot-synthesis-based peptide arrays

**DOI:** 10.1016/j.isci.2025.114498

**Published:** 2025-12-19

**Authors:** Elizabeth Leblanc, Svenja C. Schüler, Yuguo Liu, Léa Théroux, Emmeran Le Moal, Marc-André Bonin, Pierre-Luc Boudreault, C. Florian Bentzinger

**Affiliations:** 1 Département de Pharmacologie-Physiologie, Institut de Pharmacologie de Sherbrooke, Centre de Recherche Du Centre Hospitalier Universitaire de Sherbrooke, Faculté de Médecine et des Sciences de La Santé, Université de Sherbrooke, Sherbrooke, QC, Canada

**Keywords:** Biotechnology, Cell biology

## Abstract

Altered interactions with the extracellular matrix (ECM) represent a root cause of skeletal muscle stem cell (MuSC) dysfunction in aging and disease, underscoring the therapeutic potential of targeting adhesion receptors. Here, we describe the development of an approach for the medium-throughput screening of bioactive ECM-derived adhesion motifs using peptide arrays generated by highly parallel SPOT synthesis. Based on a library of ∼50 peptide sequences originating from ECM proteins, we identified several candidate motifs that robustly enhance the adhesion of MuSC-derived cells. We demonstrate that these peptide motifs can improve the *in vitro* phenotype of MuSC-derived cells isolated from dystrophic muscle and provide proof-of-concept that they can be chemically modified for efficient bioconjugation, *in vivo* basal-lamina-binding, and as receptor-targeting molecular probes. Altogether, our work provides a versatile toolkit for studying MuSC adhesion and uncovers a set of functional peptide motifs with translational potential for pro-regenerative therapies in skeletal muscle disorders.

## Highlights

• SPOT peptide arrays enable screening for ECM-derived adhesion motifs
• ECM peptides support the attachment of healthy and dystrophic myogenic progenitors
• Chemical modification converts ECM peptides into adhesion receptor probes
• ECM peptides can be configured for basal-lamina binding *in vivo*

## Graphical abstract

**Figure:**
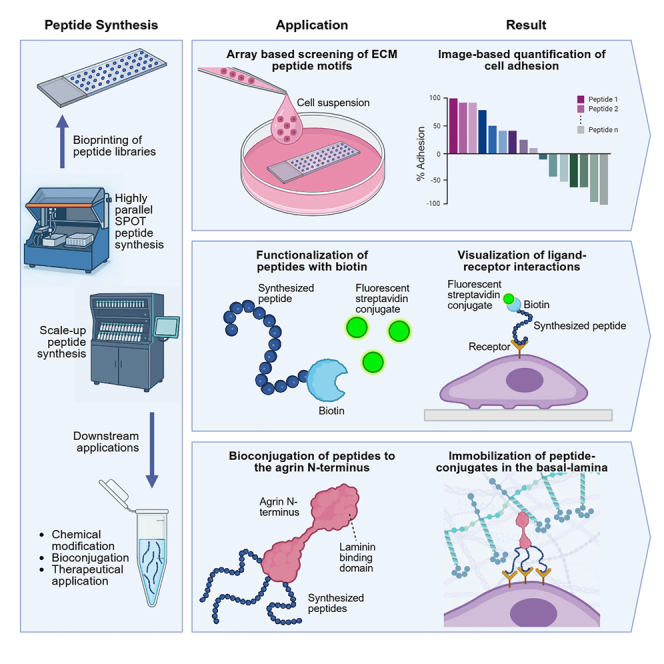

## Introduction

Under homeostatic conditions, quiescent adult skeletal muscle stem cells (MuSCs) are located in the satellite cell position in the periphery of muscle fibers, positioned beneath the extracellular matrix (ECM) of the basal-lamina.^1^^,^^2^ The basal-lamina is a sheet-like structure composed of a core network of laminin and collagen, interspersed with various other ECM components such as perlecan, biglycan, and agrin, which have functions in lateral stabilization and tethering to cell surface receptors. Several lines of evidence point toward a role of the basal-lamina in maintaining MuSC quiescence. Following injury and activation, the ECM in skeletal muscle becomes highly heterogeneous and regulates the progression of MuSCs through the myogenic program.^3^^,^^4^^,^^5^ Early after injury, the deposition of structural molecules such as fibrin and hyaluronic acid generates a microenvironment that, in conjunction with inflammatory cytokines, allows MuSCs to break quiescence and enter the cell cycle.^6^^,^^7^ Subsequently, MuSCs are exposed to a range of pro-regenerative ECM components that are not present in the homeostatic niche, including certain isoforms of laminin and fibronectin.^3^^,^^4^^,^^5^ Accompanied by the polarization of immune cells toward an anti-inflammatory state and extensive proliferation of ECM-secreting fibro-adipogenic progenitors (FAPs), the MuSC pool reaches peak expansion around 3–5 days post-injury.^8^ Skeletal muscle at this stage contains high levels of transient ECM that sets the stage for the following wave of differentiation. During MuSC lineage progression toward myocytes and their subsequent fusion into myofibers, the ECM plays an important role in sequestering pro-proliferative growth factors and in allowing the return of self-renewed stem cells to quiescence.^1^^,^^2^ Thus, under both homeostatic conditions and during all stages of skeletal muscle regeneration, a plethora of coordinated ECM signals in the niche controls MuSC function.

Conditions such as aging and muscular dystrophy are accompanied by changes in the composition of the ECM that have deleterious consequences for MuSCs. Old skeletal muscle contains lower levels of fibronectin leading to the impaired activation of integrins in aged MuSCs, which causes them to undergo anchorage dependent cell death (anoikis).^9^ Furthermore, secretion of the matricellular protein WISP1 is reduced in aged muscle leading to impaired MuSC differentiation.^10^ Therapeutic delivery of WISP1 and fibronectin, or antibody-mediated forced activation of integrin is able to significantly enhance the function of aged MuSCs and rejuvenates the regenerative capacity of skeletal muscle.^9^^,^^10^^,^^11^ Evidence from preclinical models of collagen VI-related muscular dystrophy revealed an impaired self-renewal capacity of MuSCs that seems to be linked to changes in the elasticity of skeletal muscle tissue.^12^ Intramuscular delivery of healthy fibroblasts in collagen VI-deficient mice has been shown to be therapeutically beneficial and restores MuSC function. Similar to its beneficial effects in aging, activation of integrin signaling stimulates MuSC function and slows disease progression in a mouse model of Duchenne muscular dystrophy.^11^ Moreover, skeletal muscle regeneration in mice with laminin-α2 (LAMA2)-related muscular dystrophy is severely impaired and restoring the connection to the basal-lamina using linker-molecules is able to partially reverse this phenotype.^13^ Altogether, these examples demonstrate that the aberrant ECM adhesion of MuSCs is involved in the pathogenesis of multiple skeletal muscle conditions and represents a promising therapeutic target to enhance the tissue’s regenerative capacity. However, little is known about the molecular mechanisms involved in MuSC-ECM interactions.

Here, we set out to identify and characterize critical MuSC adhesion motifs found in ECM proteins. To this end, we used highly parallel SPOT peptide synthesis in combination with a bioprinter to generate arrays for screening of ECM-derived adhesion motifs.^14^ Based on a library of ∼50 ECM peptides, we identified and characterized several adhesion motifs originating from laminin, fibronectin, vitronectin, and thrombospondin. We demonstrate that these peptides ameliorate the *in vitro* phenotype of myoblasts from mice with LAMA2-related muscular dystrophy and show that they can be chemically modified for bioconjugation, *in vivo* delivery to the basal-lamina, and as probes to visualize their target ECM receptors in myoblasts and MuSCs. Our work provides insights into the biology of MuSC-ECM interactions and identifies potential therapeutic targets for skeletal muscle conditions that are accompanied by stem cell adhesion defects.

## Results

### SPOT peptide arrays for screening of cell adhesion

**Figure 1:**
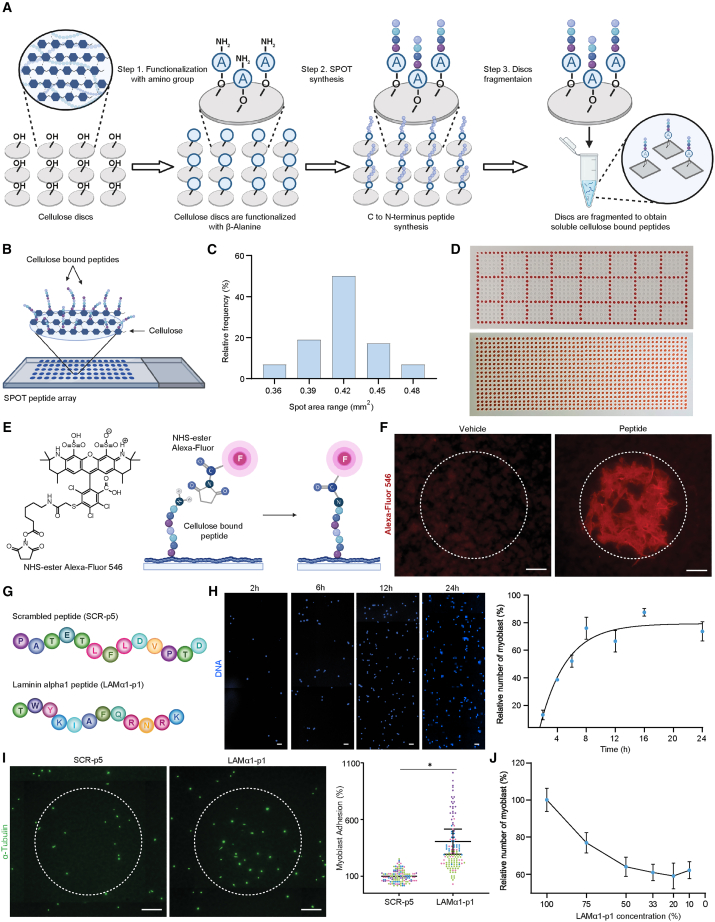
Characterization of SPOT synthesis-based peptide arrays (A) Scheme illustrates the synthesis of peptides on cellulose discs using the SPOT technique (A = β-alanine linker). (B) Illustration depicts peptides bound to a finished SPOT peptide array after bioprinting. (C) Size distribution of spots on arrays based on ink printing. *N* = 58 spots. (D) Photographs of the array layout used for the screening of cell adhesion (upper image) and a slide printed exclusively with ink (red spots, lower image). (E) Chemical structure of the fluorescent NHS-ester and scheme of the strategy used to detect peptides on printed arrays after prolonged submersion. (F) Representative images of fluorescent NHS-ester staining (red) of spots on a peptide array bioprinted with either DMSO vehicle or a peptide after 14 h of submersion in cell culture medium in a tissue culture incubator. The dashed line indicates the average spot size (0.42 mm^2^) and location based on the template layout determined using ink. Scale bar = 100 μm. (G) Amino acid sequences of the positive control peptide derived from laminin-α1 (LAMα1-p1) and a scrambled sequence (SCR-p5) that is close in length were both used for some of the subsequent experiments. (H) Representative images and adhesion kinetics of MuSC-derived myoblasts over 24 h based on Hoechst DNA staining (blue). 100% represents the number of cells initially seeded. Biological replicates from *n* = 3 mice. Data points represent means ± sem. Scale bar = 50 μm. (I) αTubulin staining (green) of myoblasts adhering on array spots printed with LAMα1-p1 or SCR-p5 after 7 h of incubation and quantification of cell numbers. *n* ≥ 20 spots from *n* = 4 mice represented by different colors in the dot-cloud. Horizontal bars represent means ± sem. *p* values were calculated with a ratio paired, one sided Student’s t test based on the average values per animal. ∗*p* < 0.05, ∗∗*p* < 0.01, ∗∗∗*p* < 0.001, ∗∗∗∗*p* < 0.0001. Scale bar = 100 μm. (J) Quantification of cell numbers with the decreasing concentration of LAMα1-p1 in bioprinted array spots. *n* ≥ 50 replicates from two myoblast lines. Data points represent means ± sem.

In order to generate ECM-derived peptide arrays that can be used to study cell adhesion, we used fluorenylmethoxycarbonyl (Fmoc)-based SPOT chemistry in a 384-well format to synthesize peptides on cellulose discs. Subsequently, cellulose discs were fragmented and printed onto a cellulose-covered standard microscope slide using a spotting robot (Figures 1A and 1B). In order to determine the spatial behavior of solutions after printing onto the cellulose membrane covered slides, we used ink to quantify the size distribution of the spots (Figure 1C). This revealed that the majority of spots reached a size of ∼0.42 mm^2^. With an estimated average area of ∼100 μm^2^ covered by a mouse MuSC-derived myoblast, a single spot of these dimensions should allow for around ∼3000 cells at full confluency. Based on this estimation, we opted for arrays containing ∼500 peptide spots for screening myoblast adhesion (Figure 1D). To demonstrate that peptides stay immobilized after prolonged exposure to a liquid environment, which would be required to let suspended myoblasts adhere to the arrays *in vitro*, we submerged slides containing printed peptide spots for 24 hours (h) in cell culture media. In order to visually assess the presence of peptides after this incubation period, we exposed the arrays to amine-reactive succinimidyl ester Alexa Fluor (NHS Alexa Fluor) (Figure 1E). Demonstrating that a significant portion of peptides remain immobilized after submersion, we observed a distinct fluorescent signal in spots containing peptide when compared to those printed with the vehicle solution alone (Figure 1F).

Laminin 111 is the main ECM component found in Matrigel, which is a gelatinous protein mixture derived from the Engelbreth-Holm-Swarm mouse sarcoma, well-known to favor the adhesion of MuSCs and myoblasts in coated polystyrene tissue culture dishes.^15^^,^^16^^,^^17^ Thus, to characterize the adhesion dynamics of myoblasts on our arrays, we used the integrin-binding peptide TWYKIAFQRNRK (LAMα1-p1) derived from laminin α1, a subunit of laminin 111, as a positive control (Figure 1G).^18^^,^^19^ Based on integrin α7 staining, our myoblast cultures were validated to contain a 95% pure population of myogenic progenitors (Figures S1A and S1B). We observed that between 12 and 16 h of culture, suspended myoblasts reach a plateau in adhesion (Figure 1H). Thus, to avoid saturation, we chose 7 h, when myoblast adhesion is roughly half-maximal, as the optimal time-point for our study. In order to visualize myoblasts on SPOT peptide arrays, we performed fluorescence imaging on a slide-scanner microscope using the highly abundant cytoskeletal protein α-tubulin as a marker for cell detection. Compared to a randomly chosen scrambled peptide (SCR-p5) of comparable length, about 4-fold more myoblasts adhered to array spots containing the LAMα1-p1 positive control 7 h after plating (Figure 1I). Notably, in a dilution series, myoblast adhesion correlated in a largely linear fashion with the LAMα1-p1 concentration used for spotting (Figure 1J). Altogether, these results support the notion that SPOT peptide arrays could serve as a medium-throughput paradigm to screen ECM peptides for their effects on myoblast adhesion.

### Identification of extracellular matrix-derived adhesion motifs

**Figure 2:**
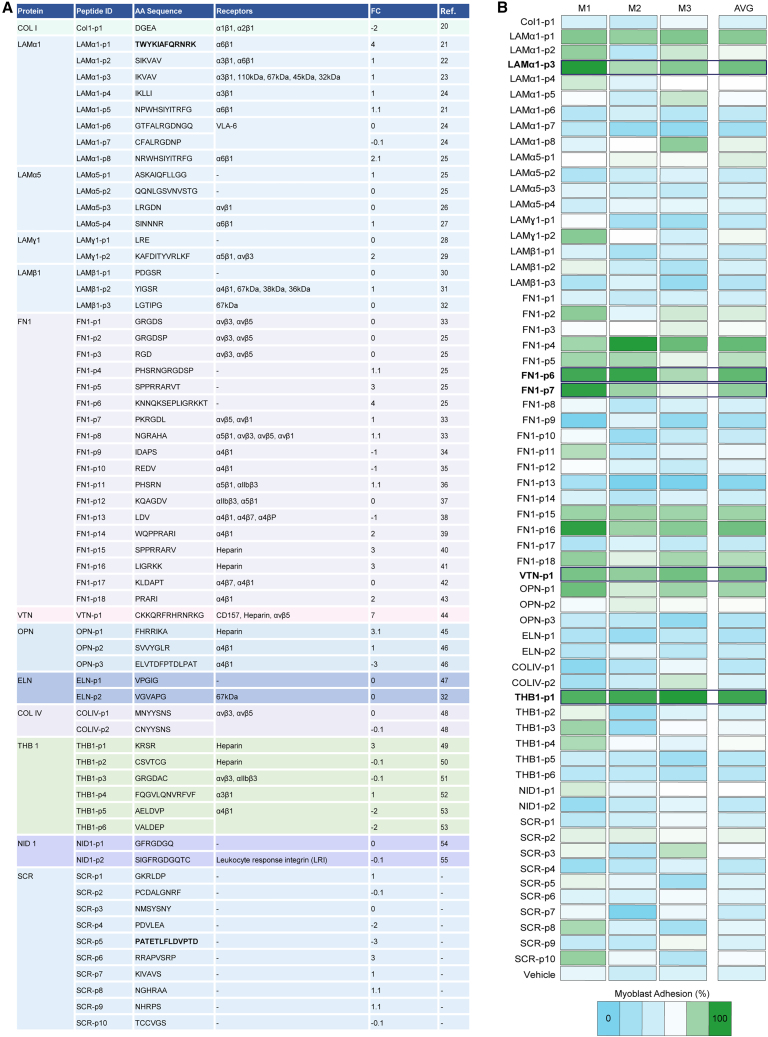
Peptide array screening of adhesion motifs (A) Table shows the library of adhesion peptide motifs used in this study. Rows contain the name of the protein of origin (protein), the abbreviation assigned to the peptides (peptide ID), the amino acid sequence (AA sequence), known cellular receptors (receptors), the focal charge of the peptides (FC), and the literature reference (Ref.). The AA sequence of the positive control peptide LAMα1-p1 is shown in bold fonts. (B) Heatmap shows the layout and number of cells adhering to peptide arrays after 7 h of incubation. Averages from *n* ≥ 6 technical replicates for each myoblast line obtained from *n* = 3 mice (M1-M3) are shown. The average (AVG) obtained from biological replicates is depicted in a separate column. Lanes with the top 5 peptides facilitating myoblast adhesion are outlined by black boxes and labeled in bold fonts.

Based on the published literature, we generated a library of 52 ECM-derived adhesion motifs (Figure 2A).^20^^,^^21^^,^^22^^,^^23^^,^^24^^,^^25^^,^^26^^,^^27^^,^^28^^,^^29^^,^^30^^,^^31^^,^^32^^,^^33^^,^^34^^,^^35^^,^^36^^,^^37^^,^^38^^,^^39^^,^^40^^,^^41^^,^^42^^,^^43^^,^^44^^,^^45^^,^^46^^,^^47^^,^^48^^,^^49^^,^^50^^,^^51^^,^^52^^,^^53^^,^^54^^,^^55^ This library contained peptides derived from collagen (COL) I and IV, laminin (LAM) α1, α5, β1, and γ1, fibronectin (FN), vitronectin (VTN), osteopontin (OPN), elastin (ELN), thrombospondin 1 (THB1), and nidogen 1 (NID1). As controls, we generated 10 different scrambled (SCR) peptides of varying lengths and amino acid composition based on selected sequences from our library. Using SPOT synthesis in conjunction with bioprinting, we subsequently generated peptide arrays covering this entire library on a single microscope slide. The peptide array slides were then submerged in culture vessels containing suspended MuSC-derived myoblasts that were detached from standard culture dishes using an enzyme-free EDTA-based dissociation buffer. After 7 h of plating, the number of adhering cells was quantified based on α-tubulin immunostaining. Across all biological replicates, this experiment revealed particularly strong adhesion for the LAMα1-derived peptide IKVAV (LAMα1-p3), the FN1-derived peptides KNNQKSEPLIGRKKT (FN1-p6) and PKRGDL (FN1-p7), the VTN-derived peptide CKKQRFRHRNRKG (VTN-p1), and the THB1-derived peptide KRSR (THB1-p1) (Figure 2B).

**Figure 3:**
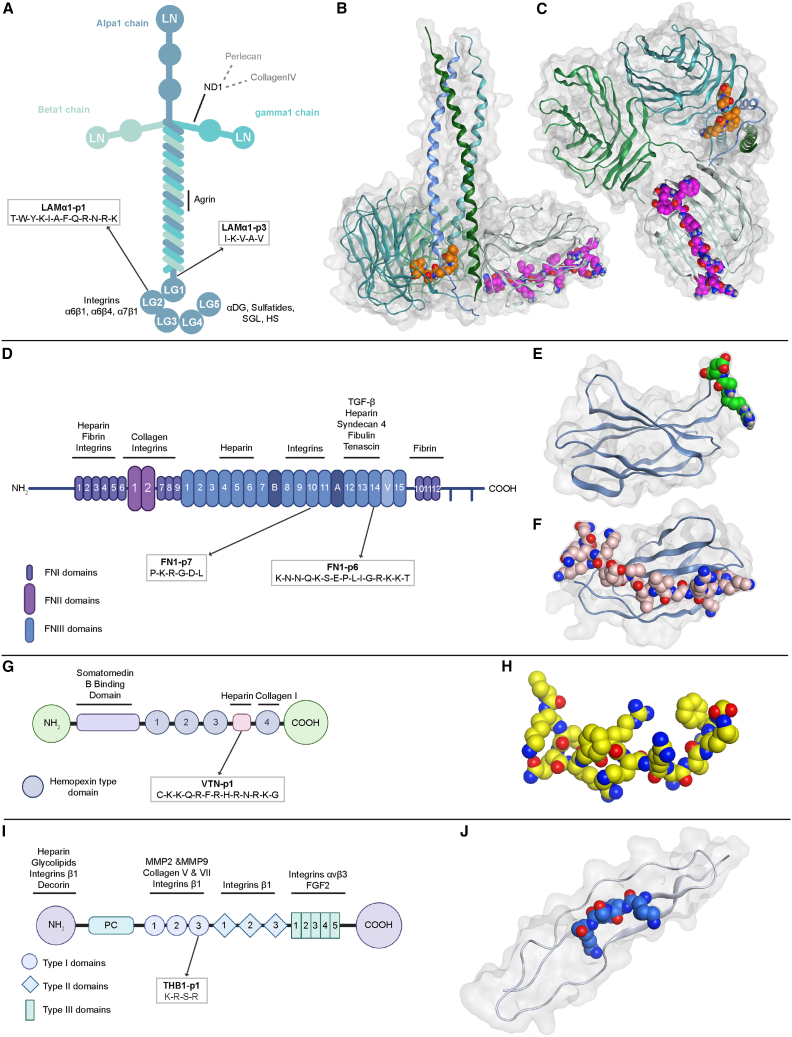
3D structure surrounding candidate peptides (A–J) Illustration of the domain structure of the extracellular matrix (ECM) molecules laminin (A), fibronectin (D), vitronectin (G), and thrombospondin (I) from which the five candidate peptides (LAMα1-p3, FN1-p6, FN1-p7, VTN-p1, and THB1-p1) originate. Sites of known binding partners are indicated, and the localization of candidate peptide sequences, as well as the positive control peptide (LAMα1-p1) are highlighted. Ribbon models with the partially transparent surface representation of published 3D structures surrounding the different candidate peptides in their ECM molecules of origin are shown on the right-hand side of the figure (laminin, PDB ID: 5MC9; fibronectin, PDB ID: 1FNF; thrombospondin, PDB ID: 1LSL).^56^^,^^57^^,^^58^ Candidate peptides are shown with the space-filling Corey-Pauling-Koltun (CPK) model in different colors. The structure surrounding VTN-p1 has not been resolved and cannot be modeled based on published information.

Distant from our positive control peptide LAMα1-p1, which is localized in the C-terminal laminin G domain-like module 2 (LG2), the peptide LAMα1-p3 maps to the carboxyl terminus of the α-helical domain of the Laminin α1 chain and is known to bind to integrin β1 (Figures 3A–3C).^59^^,^^60^ FN1-p6 localizes to the C-terminal heparin-binding 14^th^ type III repeat domain of FN1, which forms a typical β-strand and associates with proteoglycans (Figures 3D and 3E).^43^^,^^61^^,^^62^^,^^63^^,^^64^ FN1-p7 is an arginylglycylaspartic acid (RGD) containing peptide binding to integrins αvβ5, αvβ1, that is found in the 10^th^ type III repeat of FN1 as well as in other ECM molecules such as fibrinogen, vitronectin, osteopontin (Figures 3D and 3F).^33^^,^^43^^,^^56^^,^^65^^,^^66^ VTN-p1 is derived from the VTN heparin-binding domain that has not been resolved but has been demonstrated to bind to αVβ5 integrin and syndecans (Figures 3G and 3H).^44^^,^^67^ Lastly, THBS1-p1 is a proteoglycan-binding motif found in the 3^rd^ type I repeat of THBS1 and other bone-adhesive proteins such as osteopontin, bone sialoprotein, FN1, and VTN (Figures 3I and 3J).^49^^,^^68^ Taken together, these observations demonstrate that our candidate peptide motifs largely map to externally exposed surface portions of their ECM domains of origin, and they are likely to act predominantly through integrins or cell surface proteoglycans such as syndecans.

### Bioconjugation of candidate adhesion peptides for 2D culture

**Figure 4:**
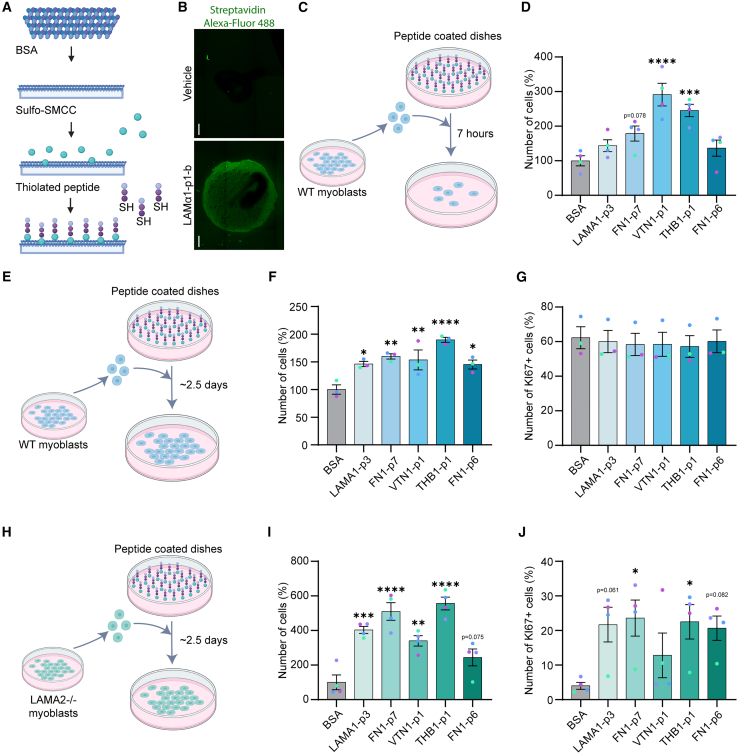
2D validation of candidate peptides (A) Scheme depicts the bovine serum albumin (BSA) coating strategy for polystyrene culture dishes with subsequent peptide functionalization. After BSA activation with sulfosuccinimidyl-4-(N-maleimidomethyl)cyclohexane-1-carboxylate (Sulfo-SMCC), C-terminally thiolated peptides were covalently linked to the coating substrate. (B) LAMα1-p1 peptides that were N-terminally linked to a biotin molecule were used for the functionalization of BSA coating as described in (A) and subsequently detected with fluorophore-coupled streptavidin (green). Serum free myoblast growth medium was used as a vehicle control. Scale bar = 500 μm. (C and D) Schematic workflow (C) and quantification (D) of WT myoblast numbers 7 h after plating on BSA-conjugated candidate peptides. Values are expressed relative to BSA coating without peptide functionalization (BSA). (E and F) Schematic workflow (E) and quantification of WT myoblast numbers (F) ∼2.5 days after plating on BSA-conjugated candidate peptides. Values are expressed relative to BSA coating without peptide functionalization (BSA). (G) Percentage of KI67 positive WT myoblasts 2.5 days after plating on BSA-conjugated candidate peptides. (H and I) Schematic workflow (H) and quantification of LAMA2−/− myoblast numbers (I) ∼2.5 days after plating on BSA-conjugated candidate peptides. Values are expressed relative to BSA coating without peptide functionalization (BSA). (J) Percentage of KI67 positive LAMA2−/− myoblasts 2.5 days after plating on BSA-conjugated candidate peptides. Horizontal bars represent means ± sem from *n* = 4 (D,I,J) and *n* = 3 (F,G) biological replicates, each corresponding to an independent myoblast line isolated from a separate mouse. *p* values relative to the unconjugated BSA control were calculated using one-way ANOVA with Dunnett correction. ∗*p* < 0.05, ∗∗*p* < 0.01, ∗∗∗*p* < 0.001, ∗∗∗∗*p* < 0.0001.

In order to functionalize polystyrene culture dishes with our candidate peptides, we used bovine serum albumin (BSA) coating to generate a proteinaceous base layer, which was activated using sulfosuccinimidyl 4-(N-maleimidomethyl)cyclohexane-1-carboxylate (Sulfo-SMCC).^69^ Candidate peptides with a C-terminal cysteine modification were then covalently linked to the activated BSA substrate through a thiol-maleimide coupling reaction (Figures 4A and S2A–S7B; Table S1). To validate this coating strategy, we functionalized the C-terminally thiolated positive control peptide LAMα1-p1 with an N-terminal biotin and used fluorescent streptavidin to visualize and confirm successful crosslinking to the BSA layer (Figures 4B and S7A and S7B). Based on this method, we subsequently quantified the adhesion of MuSC-derived wildtype (WT) mouse myoblasts 7 h after exposure to the different peptide substrates and compared it to the unconjugated BSA control. This experiment revealed significantly increased WT myoblast adhesion to the peptides VTN-p1 and THB1-p1 (Figures 4C, 4D, and S8A). We then assessed the effects of prolonged peptide exposure approximately 2.5 days after plating (Figure 4E). Extended exposure resulted in a significant increase in WT myoblast numbers, approximately 1.5– to 1.9-fold higher than in the unconjugated BSA control (Figures 4F and S8B). Since no differences were observed in the percentage of cycling KI67-positive cells, these increases were attributed primarily to enhanced cell adhesion rather than changes in proliferation (Figures 4G and S8B). To benchmark these results against a standard adhesion substrate, we quantified the number of cells adhering to collagen I relative to the unconjugated BSA control. This analysis showed an approximately 2.2-fold increase in the collagen I condition (Figures S8C–S8E), without detectable differences in the percentage of KI67-positive cells (Figure S8F).

To investigate the effects of the adhesion peptides in the context of an ECM-related pathology, we isolated myoblasts from dyW mice, a preclinical model of LAMA2-related muscular dystrophy.^70^ Loss of laminin α2 in dyW mice has been reported to disrupt basal-lamina structure, leading to muscle fiber necrosis and MuSC dysfunction.^71^^,^^72^ Interestingly, even after prolonged plating on uncoated polystyrene dishes, LAMA2−/− myoblasts isolated from dyW mice exhibited markedly reduced cell numbers compared to WT myoblasts (Figures S9A–S9C). With the exception of FN1-p6, plating on all peptide substrates significantly increased LAMA2−/− myoblast numbers, yielding approximately 3.4- to 5.6-fold higher values relative to the unconjugated BSA control (Figures 4H, 4I, and S9D). Moreover, in contrast to WT myoblasts, which showed no peptide-dependent changes in proliferation, the proportion of KI67-positive LAMA2−/−myoblasts was approximately 5.6- to 5.9-fold higher on FN1-p7 and THB1-p1 compared with the unconjugated BSA control. (Figures 4J and S9D). Comparison of collagen I with unconjugated BSA revealed an approximately 3.8-fold increase in LAMA2−/− myoblast numbers, whereas no differences were observed in the proportion of KI67-positive cells (Figures S9E–S9H). Altogether, these results establish an efficient and cost-effective strategy for peptide coating in two-dimensional culture, validate the adhesion-promoting properties of our candidate peptides in an experiment independent of the initial cellulose-based screening assay, and demonstrate that several peptides elicit distinct, disease-specific effects on dystrophic muscle progenitors that are not observed on conventional collagen I coating.

### *In vivo* delivery of basal-lamina-binding adhesion peptides

**Figure 5:**
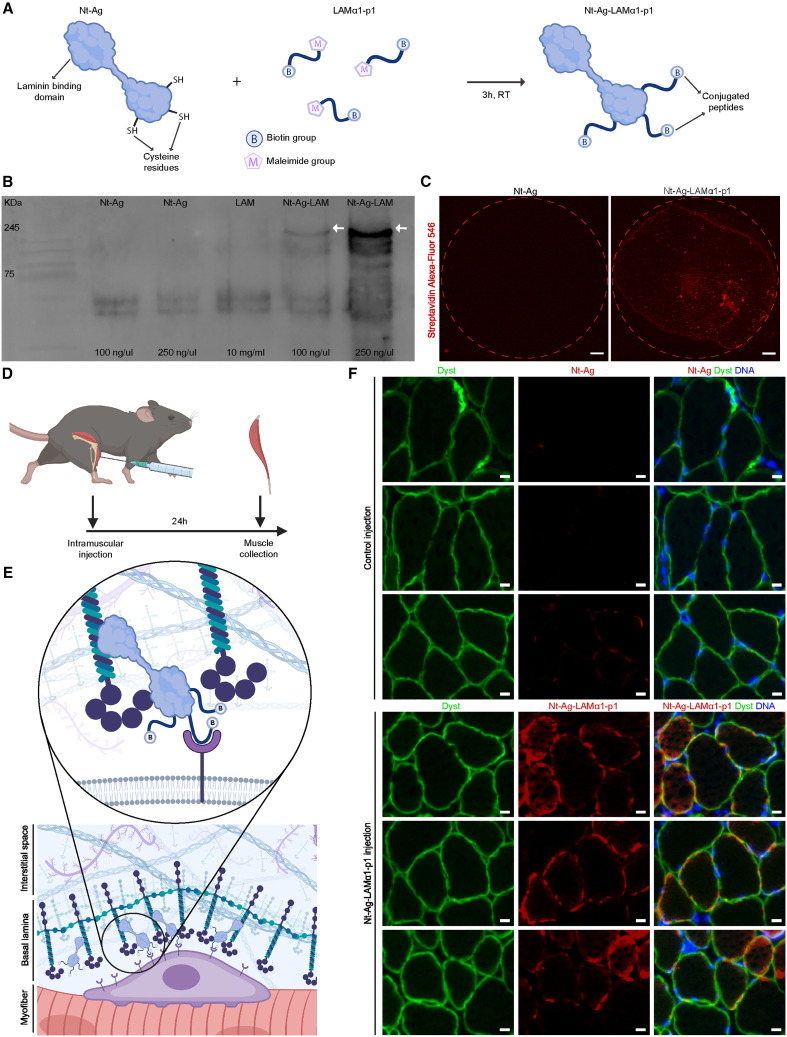
*In vivo* peptide delivery (A) Scheme shows the maleimide chemistry conjugation strategy used to couple biotinylated LAMα1-p1 peptides to the cysteines in the agrin N-terminus (NtA-Ag) to create NtA-Ag-LAMα1-p1. (B) Western blot detects biotinylated proteins using horseradish peroxidase-coupled streptavidin with two lanes containing varying amounts of NtA-Ag, one lane of LAMα1-p1, and two lanes with varying amounts of NtA-Ag-LAMα1-p1. White arrows indicate the predicted size of NtA-Ag-LAMα1-p1. (C) Detection of biotin using fluorescent streptavidin in spots containing NtA-Ag-LAMα1-p1 or Nt-Ag on laminin 111-rich Matrigel coating. Scale bars = 500 μm. (D) Experimental design and scheme show the intramuscular injection of biotinylated NtA-Ag-LAMα1-p1. (E) Illustration shows the hypothetical presentation of candidate peptides to muscle stem cells by NtA-Ag in the skeletal muscle basal-lamina after injection. (F) Dystrophin immunostaining (dyst, green) of tibialis anterior (TA) cross sections from mice injected with NtA-Ag or biotinylated NtA-Ag-LAMα1-p1 co-stained with fluorophore-coupled streptavidin (red) and Hoechst DNA staining (blue). Scale bars = 10 μm.

We next set out to develop a strategy to deliver our peptides to the basal-lamina of skeletal muscle so that they are accessible to MuSC cell surface receptors. Importantly, after injection in their solubilized form, ECM-derived peptides would be expected to function as antagonists blocking their target receptors, similar to what has been described for RGD peptides and integrins.^73^ Interestingly, the N-terminal domain of the ECM glycoprotein agrin (NtA-Ag) has a high-affinity to the coiled-coil domain of laminin and has previously been used in engineered linker-molecules in conjunction with surface receptor binding domains to increase the tethering of the basal-lamina to muscle fibers in laminin α2 deficient mice.^72^ To exploit this strategy for our purposes, we synthesized LAMα1-p1 with a C-terminal maleimide group and, for subsequent detection purposes, also modified it with an N-terminal biotin molecule (Table S1, Figures S10A and S10B). We then let the maleimide-activated peptide react with cysteine residues in purified NtA-Ag protein (Figure 5A). Western blot analysis using horseradish peroxidase-coupled streptavidin for detection demonstrated efficient crosslinking of LAMα1-p1 to NtA-Ag generating NtA-Ag-LAMα1-p1 (Figure 5B). To demonstrate that the binding capacity of NtA-Ag-LAMα1-p1 was preserved, we spotted the protein onto a 2D layer of Matrigel whose principal component is laminin 111, and detected the protein-peptide conjugate using fluorescent streptavidin. This experiment revealed that NtA-Ag-LAMα1-p1 efficiently bound to the laminin-rich substrate, while no signal was detected in the NtA-Ag control (Figure 5C). As a next step, we injected NtA-Ag-LAMα1-p1 into the *tibialis anterior* muscle of mice and compared it to NtA-Ag as a control (Figure 5D). Detection using fluorescent streptavidin revealed substantial binding of NtA-Ag-LAMα1-p1 to the basal-lamina in the periphery of dystrophin positive muscle fibers, while no signal was detected in the control condition injected with unconjugated NtA-Ag (Figure 5E and 5F). Overall, these results provide proof-of-concept for an approach to deliver MuSC adhesion peptides to the basal-lamina *in vivo*.

### Functionalization of adhesion peptides as receptor probes

**Figure 6:**
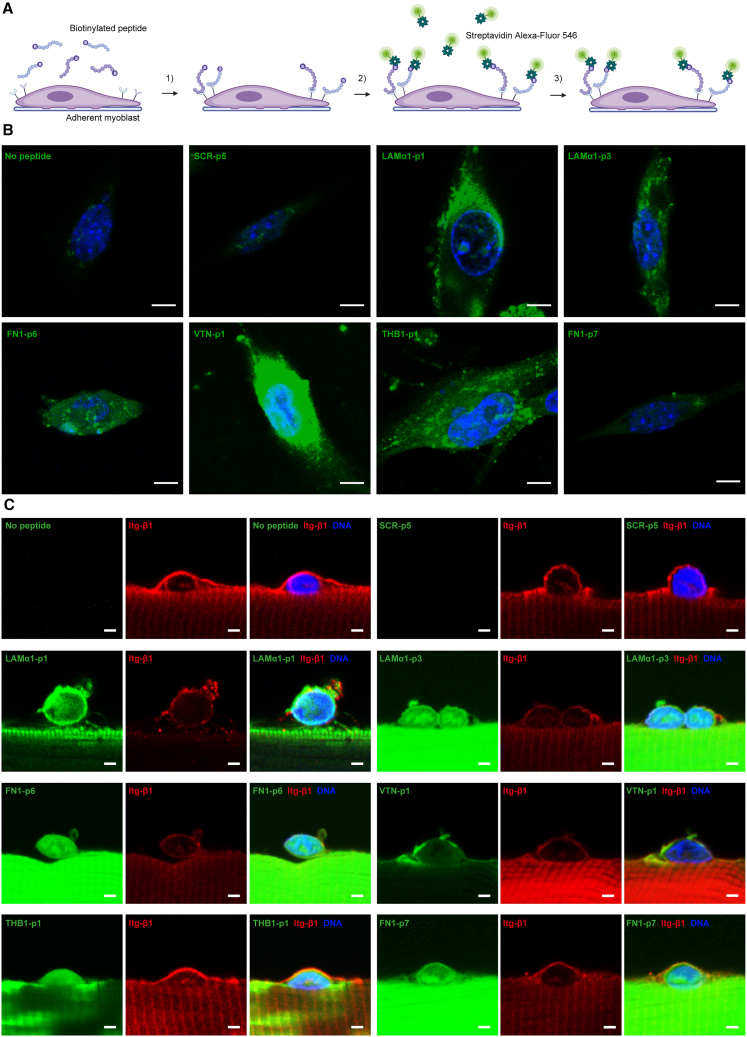
Adhesion peptides as biosensors (A) Scheme shows the detection strategy of biotinylated candidate adhesion peptides to visualize their cell surface binding. (B) Fluorescent streptavidin detection (green) of the different biotinylated candidate peptides bound to myoblasts in 2D culture in combination with Hoechst DNA staining to visualize DNA (blue). Myoblasts not incubated with peptide (No peptide) or exposed to the scrambled peptide sequences (SCR-p5) are shown as a control. Scale bar = 5 μm. (C) Integrin β1 (Itg-β1) immunostaining (red) of muscle stem cells (MuSCs) on single muscle fibers after 48 h of culture in combination with the fluorophore-coupled streptavidin detection of the different biotinylated candidate peptides (green) and Hoechst DNA staining (blue). Single fibers not incubated with peptide (No peptide) as well as one of the scrambled peptide sequences (SCR-p5) are shown as a control. Scale bar = 2 μm.

To demonstrate an additional application for our candidate peptides, we used their N-terminally biotinylated form as probes for the visualization of their target cell surface receptors (Figures 6A and S11A–S16B; Table S1). To this end, we exposed living myoblasts in traditional 2D culture on collagen I coating for 15 min to the biotinylated candidate peptides. Fluorescent streptavidin staining revealed that all candidate peptides bound in varying degrees to the myoblast plasma-membrane, while little signal was observed without peptide or in the SCR control (Figure 6B). VTN-p1 showed the strongest signal in this assay, suggesting that the potency of this peptide in mediating myoblast adhesion in 2D culture is mediated by high levels of target receptor expression. To confirm binding to MuSCs, we also exposed cultured *extensor digitorum longus* (EDL) single muscle fibers with activated MuSCs for 15 min to the different biotinylated peptides. While a significant background in muscle fibers was observed, all peptides bound to MuSCs when compared to the no-peptide or SCR control (Figure 6C).

In summary, our study demonstrates that SPOT-synthesis-based peptide arrays can be used for the medium-throughput screening of adhesion motifs for myogenic cells. We identified several promising ECM-derived candidate peptides and validated them using myogenic cells from healthy and dystrophic mice. With a bioconjugation-based method for *in vivo* delivery to the basal-lamina and a biochemical assay for the visualization of target receptors, we provide two different examples of downstream applications for these peptides. In the long run, our work may help to devise strategies for targeting adhesion signaling in MuSCs in aging and muscular dystrophy.

## Discussion

MuSC derived myoblasts are routinely cultured on collagen I-coated polystyrene dishes.^17^ However, *in vivo,* activated MuSCs, which share many characteristics with primary myoblasts, are spatially separated from the interstitial space containing fibrous collagens such as collagen I by the basal-lamina.^1^ Importantly, MuSC-derived myoblasts in collagen I coated culture dishes acquire a rounded morphology marked by membrane blebbing, a phenotype not observed when cultured on laminin.^74^ These observations support the notion that alternative coating strategies that better recapitulate basal-lamina cues may help to preserve a more representative phenotype in MuSC-derived myoblasts *in vitro*. Our study identified several efficient adhesion peptides that could potentially be exploited as affordable and effective alternatives for myoblast culture. In particular, our positive control, the laminin α1 derived peptide TWYKIAFQRNRK (LAMα1-p1), the thrombospondin derived peptide KRSR (THB1-p1), as well as the vitronectin derived peptide CKKQRFRHRNRKG (VTN-p1), showed a remarkable efficiency in promoting myoblast adhesion over both short and extended time frames post-plating. While LAMα1-p1 has been shown to engage integrins, THB1-p1 is a heparan-sulfate-binding peptide, and VTN-p1 is found in the heparin-binding domain of vitronectin, suggesting that the latter two act through cell surface proteoglycans such as syndecans.^21^^,^^44^^,^^49^ Notably, staining patterns visualized using biotinylated variants of these peptides in myoblasts only partially overlapped, indicating that they may bind distinct receptors or integrin heterodimer combinations. Beyond their performance in supporting adhesion, these peptides offer several conceptual advantages over conventional biological substrates. In contrast to collagen I, which is typically derived from rat tail tendon, or Matrigel, which is produced from a mouse sarcoma and contains a complex and variable mixture of extracellular matrix components, the peptides described here are fully synthetic and chemically defined. As such, they provide a reproducible and mechanistically interpretable platform to study cell-matrix interactions. Moreover, whereas collagen I and other natural matrices likely engage a broad range of adhesion receptors, these extracellular matrix derived peptides are expected to interact with a more restricted and defined set of membrane receptors, enabling more precise investigation of receptor specific adhesion and signaling mechanisms. Taken together, LAMα1-p1, THB1-p1, and VTN-p1 emerge as particularly promising candidates for modulating myogenic progenitor adhesion, and future studies aimed at identifying their precise receptors will be essential for uncovering the underlying signaling mechanisms.

Our study demonstrates that the maleimide chemistry-based bioconjugation of adhesion peptides using activated BSA represents a cost-effective strategy to coat standard polystyrene dishes for myoblast culture. Compared to cellulose-based peptide arrays, certain BSA-conjugated peptides showed differential effectiveness for WT myoblast adhesion at early time points post-plating. However, all candidate peptide motifs ultimately increased WT myoblast numbers after prolonged exposure. Given that the percentage of proliferating KI67-positive cells remained consistent across all conditions, these observations suggest that the adhesion kinetics of WT myoblasts are slower on some BSA-conjugated substrates compared to the cellulose-based arrays. We also observed that LAMA2−/− myoblasts from dystrophic dyW mice showed markedly improved adhesion on all BSA-conjugated peptides, except on FN1-p6. Compared to WT myoblasts, fewer LAMA2−/− cells adhered in the unconjugated BSA control in this experiment. This phenomenon led to generally increased relative effect sizes of the different BSA-conjugated peptides on LAMA2−/− cell numbers surpassing WT myoblasts two-to 5-fold. Moreover, while no effect was observed on WT cells, FN1-p7 and THB1-p1 significantly stimulated the number of KI67-positive LAMA2−/− myoblasts, suggesting that differences in proliferation or survival may also contribute to the observed effects. These findings support the notion that MuSCs in laminin α2 deficient muscular dystrophy are particularly responsive to exposure to specific extracellular matrix derived peptide motifs, likely reflecting adaptations to their pathologically altered microenvironment. More broadly, the use of defined ECM derived sequences to modulate cell behavior may offer new opportunities to influence MuSC function and improve regenerative outcomes in ECM-related myopathies and muscular dystrophies.

Skeletal muscle aging is accompanied by a loss of pro-regenerative ECM components such as WISP1 and the glycoprotein fibronectin, while integrin β1 exhibits aberrant and reduced activation patterns in aged MuSCs.^9^^,^^10^^,^^11^ Therapeutic delivery of WISP1 or fibronectin proteins, as well as the activation of integrin β1 using conformation-altering antibodies, improves aged muscle regeneration and MuSC function. Unfortunately, ECM components are often highly insoluble and present significant challenges for therapeutic use. In addition, ECM-based therapeutic molecules must be delivered across the basal- lamina to reach MuSCs. Use of the laminin-binding agrin NtA as a delivery vehicle addresses this limitation, enabling the immobilization and targeted presentation of adhesion peptides within the basal- lamina. It has previously been shown that a transgene coding for a linker molecule termed mini-agrin, which is composed of the agrin NtA and its C-terminal dystroglycan-binding domain, is able to rescue muscular dystrophy in laminin α2 mutant mice by the reanchoring of the basal-lamina to the muscle fiber plasma membrane.^72^ Importantly, mini-agrin was also found to enhance muscle regeneration in laminin α2 deficient mice, which implies that this strategy mobilizes dystrophic MuSCs.^13^ Therefore, conjugation to the agrin NtA represents a promising delivery strategy for MuSC adhesion peptides to the basal-lamina.

In recent years, hydrogel-based stem cell culture systems have been increasingly recognized as a method for *in vitro* modeling of microenvironmental interactions.^75^ Importantly, hydrogels that closely match the elasticity of native skeletal muscle tissue have been shown to better preserve stem cell characteristics in MuSC-derived cells after isolation, compared to more rigid substrates.^76^ Thus, hydrogel-based peptide arrays could serve as a powerful approach for screening cellular readouts such as self-renewal, proliferation, and differentiation of myogenic cells under physiologically relevant biomechanical conditions. Interestingly, recent work has also uncovered a role for ECM-derived signals in regulating MuSC quiescence. For instance, cell-autonomous collagen V induced by notch signaling may be involved in maintaining MuSC quiescence through the calcitonin receptor.^77^ Similarly, the activation of the G-protein-coupled adhesion receptor Gpr116 by as-yet unidentified ECM ligands has been shown to prevent cell cycle entry in MuSCs through β-arrestin1 signaling.^78^ Moreover, the transmembrane heparan sulfate proteoglycan syndecan-3 plays a key role in maintaining MuSCs in a non-proliferative, quiescent state. These findings suggest that mapping the functional domains of quiescence-promoting ECM components could inform the rational design of synthetic peptides, enabling array-based screening platforms using myogenic cells. The development of quiescence-promoting peptides would open fundamentally novel avenues for studying MuSC biology and might also lead to therapeutic strategies for conditions characterized by premature stem cell activation or aberrant differentiation.

Altogether, we demonstrate the feasibility of the medium-throughput screening of adhesion peptides using SPOT synthesis in conjunction with bioprinting. We identified several candidate motifs that stimulate myogenic progenitors from both healthy and diseased muscle, established a method for delivering peptides to the skeletal muscle basal-lamina, and showed that these peptides can be used as molecular probes to identify their respective target receptors. Our study represents a foundational step toward the detailed molecular characterization of adhesion signaling in MuSCs and may contribute to the development of future therapeutic strategies targeting regenerative dysfunction in aging and disease.

### Limitations of the study

Although this study establishes a robust and versatile platform for the identification and functional validation of ECM-derived adhesion peptides, several limitations and outstanding questions remain. Our work was performed using mouse cells, which enabled efficient *in vivo* proof-of-concept studies. However, the approach developed here could be readily extended to human MuSCs and myoblasts, which would further facilitate translation. In addition, the screening strategy was based on a candidate approach guided by ECM motifs reported in the literature. While this enabled focused the interrogation of known functional sequences, unbiased peptide discovery approaches may reveal additional adhesion motifs with biological relevance. Finally, although we demonstrate the basal-lamina targeting of peptides via intramuscular injection, local delivery of therapeutic proteins to skeletal muscle remains challenging, as not all clinically relevant muscles can be efficiently accessed, including the diaphragm. Gene therapy-based strategies enabling sustained *in situ* expression of peptide conjugates or linker molecules may represent a potential solution to overcome these limitations.

## Resource availability

### Lead contact

Requests for further information and resources should be directed to and will be fulfilled by the lead contact, C. Florian Bentzinger (cf.bentzinger@usherbrooke.ca).

### Materials availability

This study did not generate new unique reagents.

### Data and code availability

• Data reported in this article will be shared by the lead contact upon request.
• This article does not report original code.
• Any additional information required to reanalyze the data reported in this article is available from the lead contact upon request.

## Acknowledgments

C.F.B. was supported by the 10.13039/100022992Canadian Institutes of Health Research (CIHR, PJT-162442 and PJT-507167), the 10.13039/501100000038Natural Sciences and Engineering Research Council of Canada (NSERC, RGPIN-2017-05490 and RTI-2025-00383), the 10.13039/501100020951Fonds de Recherche du Québec - 10.13039/100012013Santé (FRQS, Dossiers 296357, 34813, 36789 and 366054), the Fonds de Recherche du Québec - Nature et Technologies (FRQNT, Dossier 331297), the 10.13039/501100000098ThéCell Network (supported by the FRQS), the 10.13039/501100000098Canadian Stem Cell Network, and a research chair of the Centre de Recherche Médicale de l’Université de Sherbrooke (CRMUS). P.L.B was supported by 10.13039/501100000038NSERC (RGPIN-2022-04028 and RTI-2020-00560) and 10.13039/100021557CIHR (PJT-480163). E.L.M. was supported by a postdoctoral fellowship of the 10.13039/100008240FRQS (Dossier 258477). Y.L. was supported by a 10.13039/501100000223Muscular Dystrophy Canada Research Fellowship in collaboration with the Neuromuscular Disease Network for Canada. S.C.S. was supported by a postdoctoral fellowship from the 10.13039/501100001659German Research Foundation (DFG, 505064275). Graphical illustrations in some figures were created using BioRender.com.

## Author contributions

C.F.B. and P.L.B. initiated and managed the project. E.L., S.C.S., E.L.M., and Y.L. designed and conducted experiments. L.T. carried out solid-phase peptide synthesis. M.A.B. provided support with peptide synthesis and bioprinting. C.F.B., P.L.B, E.L., and S.C.S. interpreted results and wrote the article.

## Declaration of interests

The authors declare no competing interests.

## STAR★Methods

### Key resources table

**Table:** 

REAGENT or RESOURCE	SOURCE	IDENTIFIER
**Antibodies**		

Biotin rat anti-Cd106 (VCAM)	BioLegend	Cat#105704; RRID:AB_313205
Mouse anti-α-tubulin clone DM1A	Sigma-Aldrich	Cat#T9026; RRID:AB_477593
Rat anti-CD29 (integrin-β1)	BD Bioscience	Cat#553715; RRID:AB_395001
Rat anti-KI67 (SolA15)	eBioscience	Cat#14-5698-82; RRID:AB_10854564
Rat anti-ITGA7	UBC ab lab	Cat#67-0010; RRID:AB_2890939
Rabbit anti-dystrophin	Abcam	Cat#ab15277; RRID:AB_301813
Alexa-Fluor 488 goat anti-rat (H+L)	Invitrogen	Cat#A11006; RRID:AB_2534074
Alexa-Fluor 647 goat anti-rat (H+L)	Invitrogen	Cat#A21247; RRID:AB_141778
Alexa-Fluor 488 goat anti-mouse IgG1(y1)	Invitrogen	Cat#A21121; RRID:AB_2535764
Alexa-Fluor 546 goat anti-rabbit IgG (H+L)	Sigma-Aldrich	Cat#A-11035; RRID:AB_2534093

**Chemicals, peptides, and recombinant proteins**		

Streptavidin Alexa-Fluor 546	Invitrogen	Cat#S11225; RRID:AB_2532130
Alexa-Fluor 647 Streptavidin	Sigma-Aldrich	Cat#S21374
Alexa Fluor 546 NHS Ester (Succinimidyl Ester)	Thermo Fisher	Cat#A20002
Human recombinant N-terminal agrin protein	R&D systems	Cat#8909-AG-050
Peptide sequences are detailed in Table S1	This paper	customized

**Experimental models: Organisms/strains**		

Mouse: C57Bl/6	Charles River	Strain 027
Mouse: Lama2-/- (dyW) mice (B6.129S1(Cg)-Lama2tm1Eeng/J)	The Jackson Laboratory	Strain 013786

**Software and algorithms**		

GraphPad Prism v9	GraphPad	RRID: SCR_002798
Fiji	Schindelin et al.^79^	https://imagej.net/software/fiji/
Zen v 3.4	Zeiss	https://www.zeiss.com/microscopy/en/products/software/zeiss-zen-lite.html
Molecular Operating Environment software (MOE 2024.0601)	Chemical Computing Group	https://www.chemcomp.com/en/Products.htm

### Experimental model and study participant details

Experimental work with mice was performed in accordance with the guidelines and regulations established by the animal committee of the Université de Sherbrooke, which are based on the guidelines of the Canadian Council on Animal Care, under the protocol 2020-2523. Approximately equal proportions of male and female C57BL/6 (Charles River, strain 027) or Lama2-/- (dyW, JAX 013786) mice between 4 and 6 weeks of age were used for muscle stem cell isolation.

### Method details

#### Muscle stem cell isolation

Hindlimb muscles of C57BL/6 (Charles River, strain 027) or Lama2-/- (dyW, JAX 013786) mice were dissected and washed in phosphate-buffered saline (PBS 311-010-CL, Wisent) with 1% penicillin-streptomycin solution (P/S,450-201-EL, Wisent). Muscles were minced using dissection scissors and enzymatically digested with collagenase B (0.01 g/ml, 11088831001, Roche) and dispase II (0.4%,04942078001, Roche) solution at 37°C for 30 minutes. The muscle slurry was triturated using a 5 ml pipette and pressed through a syringe with a 20-gauge needle (305175, BD). The slurry was then filtered through a 40 μm sterile cell strainer and washed with 30 ml of Dulbecco’s modified Eagle’s medium (DMEM, 319-005-CL, Wisent) with 10% fetal bovine serum (FBS, 080-450, Wisent). The resulting solution was centrifuged, and the pellet was incubated with biotin anti-mouse CD106 (VCAM1) antibody (1:200, 105704, BioLegend) for 1h on ice. After washing, the pellet was incubated with MACS streptavidin beads solution (1:200, 130-048-101, Miltenyi Biotec) for 1 h on ice. VCAM1+ cells were sorted using magnetic-activated cell sorting (MACS, 130-042-301, Miltenyi Biotec) with an LS column (130-042-401, Miltenyi Biotec). The cells were collected in collagen I (C3867-1VL, Sigma-Aldrich) coated polystyrene culture dishes (83.3902, Sarstedt) with growth medium containing Ham’s F10 (318-050-CL, Wisent) supplemented with 20% FBS, 1% P/S and 2.5 ng/ml of basic fibroblast growth factor (bFGF, 10821-962, VWR) and maintained in a 5% CO_2_ incubator at 37°C.

#### Peptide arrays

Peptides were synthesized on a ResPep SLi Peptide Synthesis unit (Intavis) using fluorenylmethoxycarbonyl (Fmoc) based chemistry on β-alanine functionalized cellulose discs (32.121, CEM) according to the manufacturer’s protocol. Briefly, Fmoc-protected amino acid solutions were dissolved in N,N-Dimethylformamide (DMF, 039117.M6, Thermo Fisher) at a concentration of 0.5M. Membranes were deprotected twice with 20% piperidine (104094, Sigma-Aldrich) in DMF. The membranes were washed with DMF followed by Ethanol (EtOH, 044134.AE, Thermo Fisher). Preactivation of the Fmoc-protected amino acid was performed using Oxyma pure (3849-21-6, Matrix Innovation), Diisopropylcarbodiimide (DIC, A19292.18, Thermo Fisher), and N-methylpyrrolidone (NMP, N140-1, Thermo Fisher). Each amino acid was coupled twice for 20 minutes. Following coupling, N-terminal amino acids were capped with a 5% acetic anhydride (Ac2O, 1000631011, Sigma-Aldrich) solution in DMF. After capping, the membranes were washed with DMF. Deprotection, activation, coupling, and capping cycles were repeated until the complete peptide sequence was obtained. Once the last amino acid was added, the membranes were deprotected with 20% piperidine solution in DMF and washed with DMF followed by EtOH. Evaporation was performed for 750 seconds under vacuum. No final capping step was performed to leave the N-terminal amino acid free. Peptide-bound membranes were incubated with the side chain cleavage solution composed of 80% trifluoroacetic acid (TFA, 00289, Chem-Impex), 3% triisopropylsilane (TIPS, 233781, Sigma-Aldrich), 5% H_2_O, and 12% dichloromethane (DCM, 650463, Sigma-Aldrich) for 2 hours in a fume hood. Cellulose discs with peptides were then incubated in the main cleavage solution composed of 88.5% TFA, 4% trifluoromethanesulfonic acid (TFMSA, 158534, Sigma-Aldrich), 2.5% TIPS and 5% H_2_O, overnight at room temperature allowing them to dissolve completely. Ice-cold methyl *tert*-butyl ether (MTBE, 306975, Sigma-Aldrich) was added to each tube and placed at -20°C for 1 h. The peptides were centrifuged for 5 minutes at 8,160 g to compact the precipitate. Pellets were washed twice with ice-cold MTBE, dried for 10 minutes, dissolved in 500 μl dimethylsulfoxide (DMSO, 472301, Sigma-Aldrich), and tubes containing the peptides were stored at -20°C. Cellulose-bound peptide solutions were thawed and loaded into 384-well Intavis plates (54080, CEM). A Slide Spotting Robot (Intavis) was set to dispense a needle volume of 0.05 μl for each spot and to do two rinsing and dispensing cycles between each peptide. The device was set to spot an ink grid before the peptides with the same dispenser volume. After spotting the peptide arrays were left to dry for 2 hours in a 37°C oven and then stored at 4°C in the dark until needed. Spot size analysis was performed using images taken by an AxioImager M2 Apotome (Zeiss) using the Zen software (Zeiss). Results were visualized using GraphPad Prism.

#### Myoblast adhesion on peptide arrays

Primary mouse myoblasts were dissociated from collagen I coated culture dishes using enzyme-free dissociation buffer (13151014, Gibco) and resuspended at a concentration of 10000 cells / cm^2^ in growth medium. For cell seeding peptide arrays were placed in a 10 cm polystyrene culture dish and the cells were allowed to adhere for 7 hours. For quantification, the slides were PBS washed, fixed with 4% paraformaldehyde (PFA, 416785000, Thermo Fisher) for 10 minutes, and washed with PBS. The slides were incubated for 10 minutes with 0.5% Triton-X-100 (TB0198, Bio Basic) in PBS followed by PBS washing. For blocking, the slides were incubated in 5% bovine serum albumin (BSA, 5217, Tocris Bioscience) in PBS solution for 1h at room temperature. Excess solution was removed, and the slides were incubated with monoclonal anti-α-tubulin mouse primary antibody (T9026, Sigma-Aldrich, 1:500) in 5% BSA in PBS at 4°C overnight. The slide was then PBS washed and incubated with Alexa-Fluor 488 goat anti-mouse IgG1 (1:500, A21121, Invitrogen) in 5% BSA in PBS for 1h at 37°C. The sample was PBS washed and mounted with Fluoromount-G (00-4958-02, Invitrogen). Stainings were visualized with an AxioImager M2 Apotome (Zeiss), cells were quantified using FIJI,^79^ and visualization was performed using GraphPad Prism.

#### Retention of peptides on submerged arrays

Peptide arrays were placed in a 10 cm polystyrene culture dish and submerged in myoblast growth medium containing Ham’s F10, 20% FBS, 1% P/S and 2.5 ng/ml bFGF and maintained in a 5% CO_2_ incubator at 37°C for 24 h. After removal of growth medium, the slide was washed with PBS. The arrays were incubated in a solution of Alexa Fluor 546 NHS Ester (Succinimidyl Ester) dye (A20002, Thermo Fisher) at a concentration of 2 μg/ml in PBS at 37°C for 1 h. The slide was washed with PBS and mounted with Fluoromount-G. The staining was visualized with an AxioImager M2 Apotome (Zeiss).

#### Solid phase peptide synthesis

Peptides were synthesized at a 0.1 mmol scale on 2-chlorotrityl chloride resin (2-401-1310, Matrix Innovation) using Fmoc-based chemistry and a Symphony X multiplex peptide synthesizer (Gyros Protein Technologies). The resin was swelled in DCM for 5 min and then filtered. The first Fmoc-protected amino acid (0.2 mmol, Matrix Innovation) and N,N diisopropylethylamine (0.5 mmol, DIPEA, 00141, Chem-Impex) were mixed with the resin in DCM on a shaker overnight. To cap unreacted 2-chlorotrityl chloride groups, a solution of DCM/methanol/DIPEA (7/2/1) was added to the resin. The Fmoc group of the first amino acid was removed by treating with 20% piperidine in DMF and subsequent DMF washing. The second Fmoc-protected amino acid was then coupled using hexafluorophosphate azabenzotriazole tetramethyl uronium (0.5 mmol, HATU, 1-063-0001, Matrix Innovation) and Oxyma Pure (0.5 mmol) in the presence of DIPEA (1 mmol) in DMF, and the mixture was bubbled with nitrogen for 30 min. Upon completion of the reaction, the resin was washed with DMF. Deprotection and coupling steps were repeated to produce the complete desired peptide sequence. Except for compounds with D-biotin, the resin was deprotected with 20% piperidine in DMF. Finally, the resin was washed with DMF, followed by DCM, then dried under vacuum before the final cleavage. Peptides were cleaved with a mixture of TFA/TIPS/1,2-ethanedithiol (EDT, 8.00795, Sigma-Aldrich)/H_2_O (92.5/2.5/2.5/2.5) for a minimum of 2h30. One hour was added for each arginine present in peptides. The cleavage solution was filtered and added dropwise into MTBE at 0 °C to precipitate the peptide and the mixture was centrifuged to separate the crude product. For maleimide addition, the allyloxycarbonyl (Alloc) groups of LAM-α1-p1 were deprotected with tetrakis(triphenylphosphine)-palladium(0) (0.2 eq, 216666, Sigma-Aldrich) and Phenylsilane (20 eq, 335150, Sigma-Aldrich) in DCM degassed for 30 min. The resin was washed twice with a solution of sodium diethyldithiocarbamate (0.01 M, 71480, Sigma-Aldrich) in DMF for 5 min, followed by DMF. 6-Maleimidohexanoic acid (0.5 mmol, OR-2232, Combi-Blocks) was then coupled, and the resin was cleaved as described above. To add a diethylene glycol (PG2) spacer between the peptides and biotin or cysteine groups, 8-(Fmoc-amino)-3,6-dioxaoctanoic acid (07310, Chem-Impex) was used for synthesis, and coupling and deprotection were carried out as described above. The crude product was dissolved in 1 mL of DMSO (472301, Sigma-Aldrich) and 0,8 mL of 60:40 (v/v) acetonitrile (ACN, 34851, Sigma-Aldrich)/water mixture with 0.1% formic acid (Millipore Sigma, 27001). Final compounds were purified on the preparative HPLC-MS system (Waters) with a 2998 UV detector, MS SQ Detector 2, 2767 sample manager, 2545 binary gradient module, and an XSelect CSH Prep C18 (19 × 100 mm) column (186005421, Waters) packed with 5 μm particles with 130 Å pore size for acidic condition and XBridge BEH C18 (19 x 100 mm) column (186002978, Waters) packed with 5 μm particles with 130 Å pore size for basic condition. A binary solvent system with acetonitrile/water + 0.1% formic acid was used for acidic condition and 0.03% ammonium hydroxide (05002, Sigma-Aldrich) in acetonitrile with 20 mM ammonium bicarbonate (A6141, Sigma-Aldrich) aqueous solution for basic condition. Purity of compounds was evaluated using a UPLC-MS system (Waters) with an Acquity UPLC CSH C18 (2.1 × 50 mm) column (186005296, Waters) packed with 1.7 μm particles with 130 Å pore size in acidic condition (solvent A: acetonitrile with 0.1% formic acid, solvent B: water with 0.1% formic acid) or basic condition (solvent C: acetonitrile with 0.03% ammonium hydroxide, solvent D: 10 mM ammonium bicarbonate aqueous solution with 0.3% ammonium hydroxide) with the following gradient: 0 → 0.2 min: 5% acetonitrile (solvent A or C); 0.2 → 1.5 min: 5 → 95%; 1.5 → 1.8 min: 95%; 1.8 → 2.0 min: 95 → 5%; 2.0 → 2.5 min: 5%). High resolution mass spectroscopy (HRMS) data were recorded with a maXis ESI-Q-Tof. Pure product fractions were lyophilized to result in the final product. All peptides had a purity >96% (Table S1, Figures S2A–S7B and S10A–S16B), except for FN1-p6-b, which reached ∼93%.

#### Peptide probes

Myoblasts were cultured as previously described and seeded onto collagen-coated coverslips after detachment. Biotin-coupled peptides were dissolved in serum-free growth medium at a concentration of 2 mg/ml. The cells were incubated with the peptide solution for 15 minutes at 37°C in 5% CO_2_. To remove any unbound peptides, the cells were washed with PBS. Cells were fixed with 4% PFA solution for 10 min at room temperature and stained with Streptavidin Alexa-Fluor 546 (S11225, Invitrogen, 1:500) and Hoechst (H1399, Life Technologies, 1:2000) for 1h at 37°C. The coverslips were washed with PBS and mounted to a microscopy slide using Fluoromount-G. For single fiber isolation, 8-week-old wild-type C57BL/6 mice were euthanized using 5% isoflurane followed by cervical dislocation. Extensor digitorum longus (EDL) myofibers were cultured as described by Pasut et al. 2013.^80^ Briefly, EDL muscles were placed in pre-warmed collagenase type I (SCR103, Sigma-Aldrich) solution (0.2% in DMEM) at 37°C for 1 h. Pre-warmed DMEM was added and the myofibers were flushed using a glass pipette. Separated myofibers were washed and transferred into horse serum (H1270, Sigma-Aldrich) coated dishes containing DMEM (319-005-CL, Wisent) with 20% FBS and 1% P/S for 48 h at 37°C in 5% CO_2_. Biotinylated peptides were added at 2 mg/ml in serum-free DMEM for 15 minutes. Myofibers were then fixed with 4% PFA solution for 10 min, incubated with 1% glycine (GB0235, Bio Basic) in PBS and blocked for 1 h in a solution containing 5% BSA in PBS. Staining was performed with rat anti-mouse CD29 (integrin-β1) antibody (1:500, 553715, BD Bioscience) in blocking solution overnight at 4°C and then incubated for 1 h at 37°C in a solution of DAPI (1:2000, 40043, Biotium), Streptavidin Alexa-Fluor 546 (1:500, S11225, Invitrogen) and Alexa-Fluor 488 goat anti-rat (H+L) (1:500, A11006, Invitrogen). After washing, the slides were mounted with Mowiol and imaged with an LSM FV1000 (Olympus).

#### Coating and 2D culture

Culture dishes were coated with 0.1 mg/mL collagen type I (rat tail; C3867-1VL, Sigma-Aldrich) dissolved in 0.1 N acetic acid for 1 h at room temperature. The coating substrate was removed, and the culture dishes were dried at RT before use. For peptide coating culture dishes were coated with an 8mg/ml BSA solution at 37°C in 5% CO_2_ for 1 h and the dishes were washed with PBS. A solution of sulfosuccinimidyl 4-(N-maleimidomethyl) cyclohexane-1-carboxylate (Sulfo-SMCC) at a concentration of 1.8 mg/ml in phosphate buffer containing 12mg/ml monobasic sodium phosphate (331988, Sigma-Aldrich) in H_2_O adjusted to pH 7.2 was added to the dishes and incubated at room temperature for 1 h followed by PBS washing. Peptides functionalized with a C-terminal cysteine in phosphate buffer at a final concentration of 10 mM were incubated in the wells overnight at room temperature followed by washing with phosphate buffer. Myoblasts from either wild type C57BL/6 or Lama2-/- (dyW) mice were detached using enzyme-free dissociation buffer and were left to adhere for 7 h or ∼2.5 days and then fixed with PFA 4% solution for 10 minutes at room temperature. The wells were washed with PBS and permeabilized with 0.5% Triton X-100 solution for 10 minutes at room temperature followed by PBS washing. For blocking, 5% BSA in PBS was added for 1 h at room temperature and the cells were incubated overnight at 4°C in an antibody solution composed of monoclonal anti-α-tubulin mouse primary antibody (1:500, T9036, Sigma-Aldrich), integrin α7 (1:100, 67-0010, UBC ablab) or monoclonal anti-KI67 rat primary antibody (14-5698-82, eBioscience, 1:500) in blocking solution. The cells were washed with PBS and incubated with Phalloidin-iFluor 555 (1:1000, ab176756, Abcam), Hoechst (1:2000, H1399, Life Technologies) and anti-mouse Alexa-Fluor 488 goat anti-mouse IgG1 (1:500, A21121, Invitrogen),Alexa-Fluor 647 goat anti-rat IgG (H+L) (1:500, A-21247, Invitrogen) or Alexa-Fluor 488 goat anti-rat IgG (H+L) (1:500, A11006, Invitrogen) in blocking solution for 1 h at 37°C. Subsequently the cells were PBS washed and imaged using an Operetta CLS (PerkinElmer) or Cell Discoverer (Zeiss) microplate imager.

#### Peptide conjugated agrin NtA

500 μg/ml human recombinant N-terminal agrin protein (NtA-Ag, 8909-AG-050, R&D systems) and 350 μg/ml biotinylated NtA-Ag-LAMα1-p1 peptide in PBS were combined at a 1:20 ratio. The reaction was stirred for 3 h at room temperature and then dialyzed using a cassette with 10 KDa cutoff (66455, Thermo Fisher) in water under agitation for 2 h. The protein in solution was then collected, lyophilized and stored at -20 °C. Western blot characterization of NtA-Ag-LAMα1-p1 and unconjugated NtA-Ag was performed using horseradish peroxidase-coupled streptavidin (N100, Thermo Fisher). Injections into the tibialis anterior (TA) of wt mice were performed using 50 μL of 1.6 mg/ml NtA-Ag-LAMα1-p1 or unconjugated NtA-Ag in sterile PBS through the skin. 24 hours after the injection, the mice were sacrificed for histology. TA muscles were snap frozen in liquid nitrogen-cooled isopentane, sectioned, fixed, permeabilized and blocked as described above for cells, and incubated with an anti-dystrophin rabbit antibody (1:500, ab15277, Abcam,) overnight at 4°C. After PBS washing, secondary Alexa-fluor 546 anti-rabbit IgG (H+L) antibodies (1:500, A-11035, Sigma-Aldrich), Alexa-fluor 647 Streptavidin (1:500, S21374, Sigma-Aldrich); and Hoechst (1:2000) were applied for 1h at 37°C. The slides were mounted with Fluoromount-G and imaged with an AxioImager M2 Apotome microscope (Zeiss).

#### 3D modeling of candidate peptides

3D models of candidate peptides and overlay with published 3D structures were produced using the Molecular Operating Environment (MOE) software (Chemical Computing Group). Published data were obtained from the RCSB protein data bank (RCSB.org)^81^ and included the crystal structures of laminin (PDB ID: 5MC9), fibronectin (PDB ID: 1FNF), and thrombospondin (PDB ID: 1LSL).^56^^,^^57^^,^^58^ For published structures, the ribbon model with partially transparent surface representation was used, while candidate peptides were overlaid using the space-filling Corey–Pauling–Koltun (CPK) model.

### Quantification and statistical analysis

Sample sizes reported as n values in the figure legends represent biological replicates derived from independent experimental repeats using cells isolated from separate mice. Representative images were confirmed in at least three independent experiments with similar results. Experiments were designed to include cells from male and female mice in approximately equal proportions. Data were analyzed with sex as a biological variable, but no sex-dependent differences were observed. Therefore, samples and data points were pooled and treated as equivalent biological replicates. Sample size determination was based on the expected effect size and variability previously observed for similar readouts in the investigators’ laboratory. Image acquisition and quantification were performed in a blinded manner. Cell numbers were quantified in a semi-automated manner using the FIJI open-source image processing package for ImageJ. Statistical analyses were performed using GraphPad Prism version 9. Statistical significance is indicated as follows: ∗P<0.05, ∗∗P<0.01, ∗∗∗P<0.001, and ∗∗∗∗P<0.0001. Ratio-paired one-sided Student’s t tests were used for comparisons in which matched measurements from the same biological units were analyzed under two conditions. Unpaired two-tailed Student’s t tests were used for comparisons between two independent groups. One-way ANOVA was applied for experiments involving more than two groups, followed by Dunnett’s multiple-comparisons test when multiple conditions were compared to a single control, or Tukey’s multiple-comparisons test when all pairwise comparisons were performed. All statistical details, including exact tests used and sample sizes, are provided in the figure legends.
