# Supplementary material for: Identification of skeletal muscle stem cell adhesion motifs using spot-synthesis-based peptide arrays
Source: iScience. 2025 Dec 19;29(1):114498. doi: 10.1016/j.isci.2025.114498 (PMC12808901; doi:10.1016/j.isci.2025.114498)
Supplement: Document S1. Figures S1–S16 and Table S1 [file mmc1.pdf]

iScience, Volume *29*

Supplemental information

Identification of skeletal muscle stem cell adhesion motifs using spot-synthesis-based peptide arrays

Elizabeth Leblanc, Svenja C. Schüler, Yuguo Liu, Léa Théroux, Emmeran Le Moal, Marc-André Bonin, Pierre-Luc Boudreault, and C. Florian Bentzinger

ID	Peptide sequence	Chemical Formula	% Purity (UV, 210-400 nm)	Observed Ion	Calculated m/z	Observed m/z
LAMα1-p3-c	I-K-V-A-V-PG2-C	$C_{34}H_{64}N_8O_{10}S$	98	$[M+2H]^{2+}$	389.2306	389.2308
FN1-p7-c	P-K-R-G-D-L-PG2-C	$C_{38}H_{68}N_{12}O_{13}S$	96	$[M+2H]^{2+}$	467.2448	467.2457
VTN-p1-c	S-K-K-Q-R-F-R-H-R-N-R-K-G-PG2-C	$C_{80}H_{140}N_{34}O_{21}S$	99	$[M+4H]^{4+}$	487.2736	487.2748
THB1-p1-c	K-R-S-R-PG2-C	$C_{30}H_{59}N_{13}O_{10}S$	99	$[M+2H]^{2+}$	397.7187	397.7199
FN1-p6-c	K-N-N-Q-K-S-E-P-L-I-G-R-K-K-T-PG2-C	$C_{83}H_{149}N_{27}O_{27}S$	96	$[M+4H]^{4+}$	498.0282	498.0284
LAMα1-p1-b	Biotin-T-W-Y-K-I-A-F-Q-R-N-R-K-PG2-C	$C_{94}H_{145}N_{27}O_{23}S_2$	100	$[M+4H]^{4+}$	522.2691	522.2682
LAMα1-p3-b	Biotin-PG2-I-K-V-A-V	$C_{41}H_{73}N_9O_{11}S$	98	$[M+2H]^{2+}$	450.7647	450.7645
FN1-p7-b	Biotin-PG2-P-K-R-G-D-L	$C_{45}H_{77}N_{13}O_{14}S$	99	$[M+2H]^{2+}$	528.7789	528.7792
VTN-p1-b	Biotin-PG2-C-K-K-Q-R-F-R-H-R-N-R-K-G	$C_{87}H_{149}N_{35}O_{22}S$	100	$[M+5H]^{5+}$	414.8345	414.8354
THB1-p1-b	Biotin-PG2-K-R-S-R	$C_{37}H_{68}N_{14}O_{11}S$	100	$[M+3H]^{3+}$	306.5043	306.5064
FN1-p6-b	Biotin-PG2-K-N-N-Q-K-S-E-P-L-I-G-R-K-K-T	$C_{90}H_{158}N_{28}O_{28}S$	93	$[M+4H]^{4+}$	529.0460	529.0457
SCR-p5-b	Biotin-PG2-P-A-T-E-T-L-F-L-D-V-P-T-D	$C_{80}H_{124}N_{16}O_{28}S$	98	$[M+2H]^{2+}$	895.4318	895.4330
LAMα1-p1-mal	Biotin-PG2-T-W-Y-K-I-A-F-Q-R-N-R-K-Maleimide	$C_{101}H_{151}N_{27}O_{25}S$	99	$[M+3H]^{3+}$	726.0447	726.0461

Table S1. Characterization Table of Peptides Synthesized by solid phase peptide synthesis. Rows include the peptide identification name (ID), the amino acid sequence of the peptide (Peptide sequence), the chemical formula, the percent purity assessed by ultra-performance liquid chromatography-mass spectrometry, the calculated mass-to-charge ratio (calculated m/z) and the observed mass-to-charge ratio (observed m/z) of the ion by high-resolution mass spectrometry. Diethylene glycol (PG2) was added as a spacer between peptides and biotin or cysteine groups.

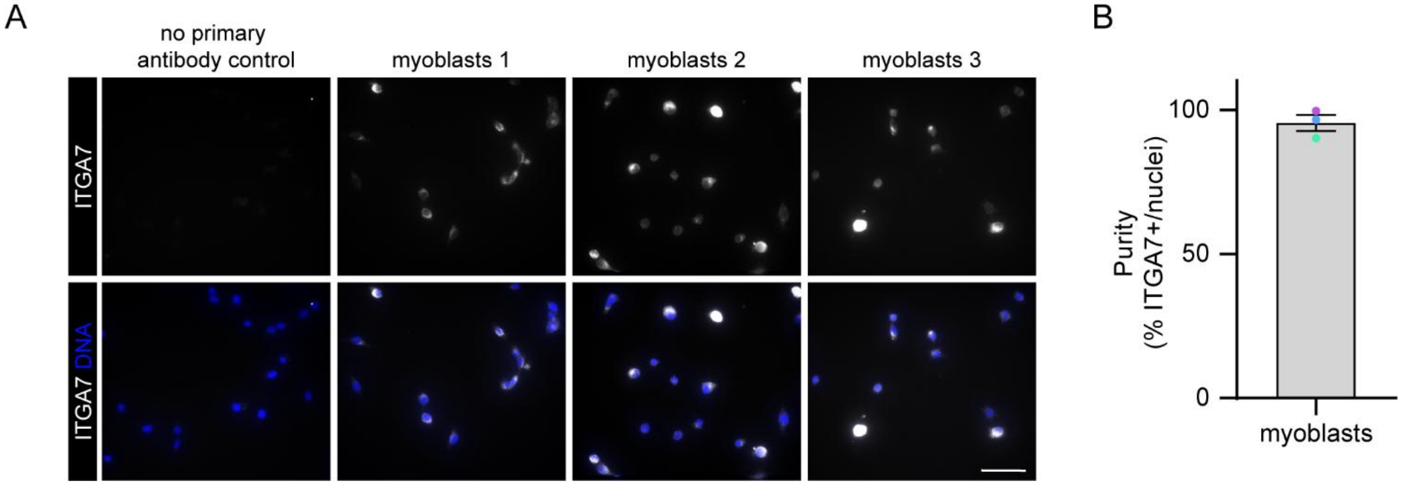

Figure S1. Purity of MuSC-Derived Myoblast Cultures. (A) Representative fluorescence images of three independent myoblast lines, each isolated from a separate mouse, stained for integrin α7 (ITGA7; white) and DNA (Hoechst; blue). A control staining in which the primary antibody was omitted is shown. Scale bar = 50 µm. **(B)** Quantification of the percentage of ITGA7 positive ($ITGA7^{+}$) myoblasts among Hoechst positive nuclei. Data represent mean ± sem from n = 3 biological replicates, each corresponding to an independent myoblast line isolated from a separate mouse.

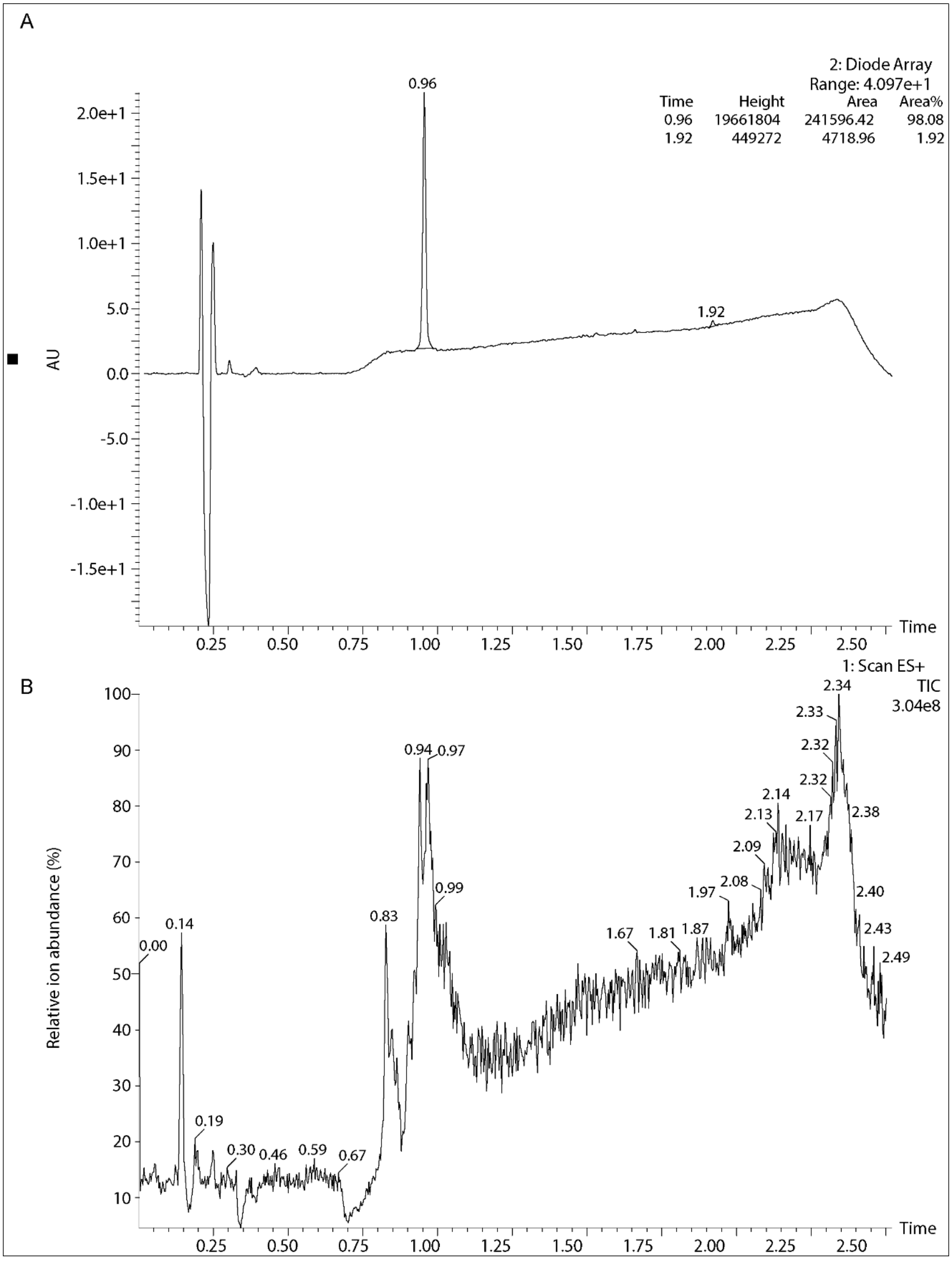

Figure S2. UPLC-MS Chromatogram for LAMα1-p3-c. (A) UV chromatogram with absorbance units (AU) plotted against time in minutes. **(B)** Mass chromatogram showing relative abundance of ions (%) plotted against time in minutes.

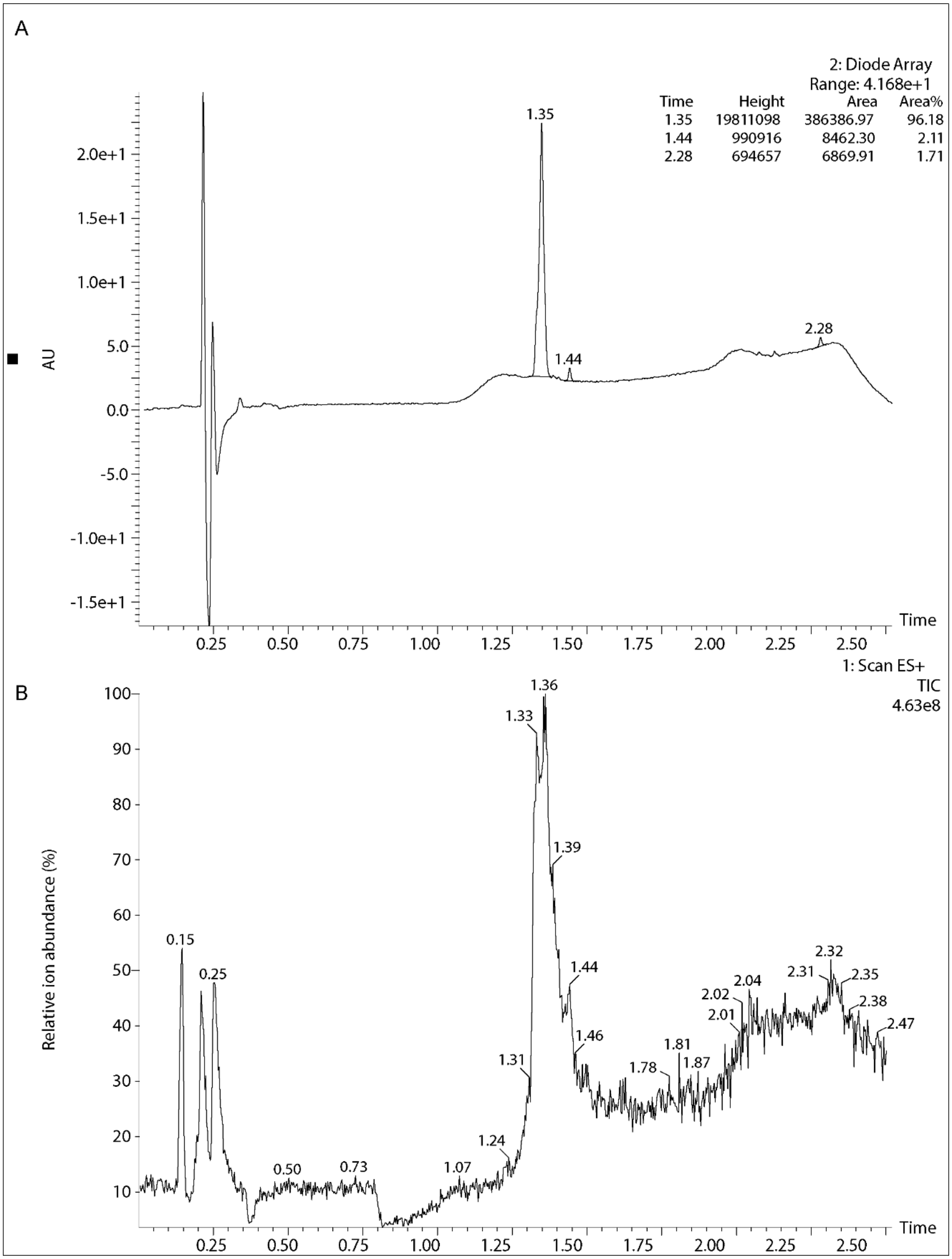

Figure S3. UPLC-MS Chromatogram for FN1-p7-c. (A) UV chromatogram with absorbance units (AU) plotted against time in minutes. **(B)** Mass chromatogram showing relative abundance of ions (%) plotted against time in minutes.

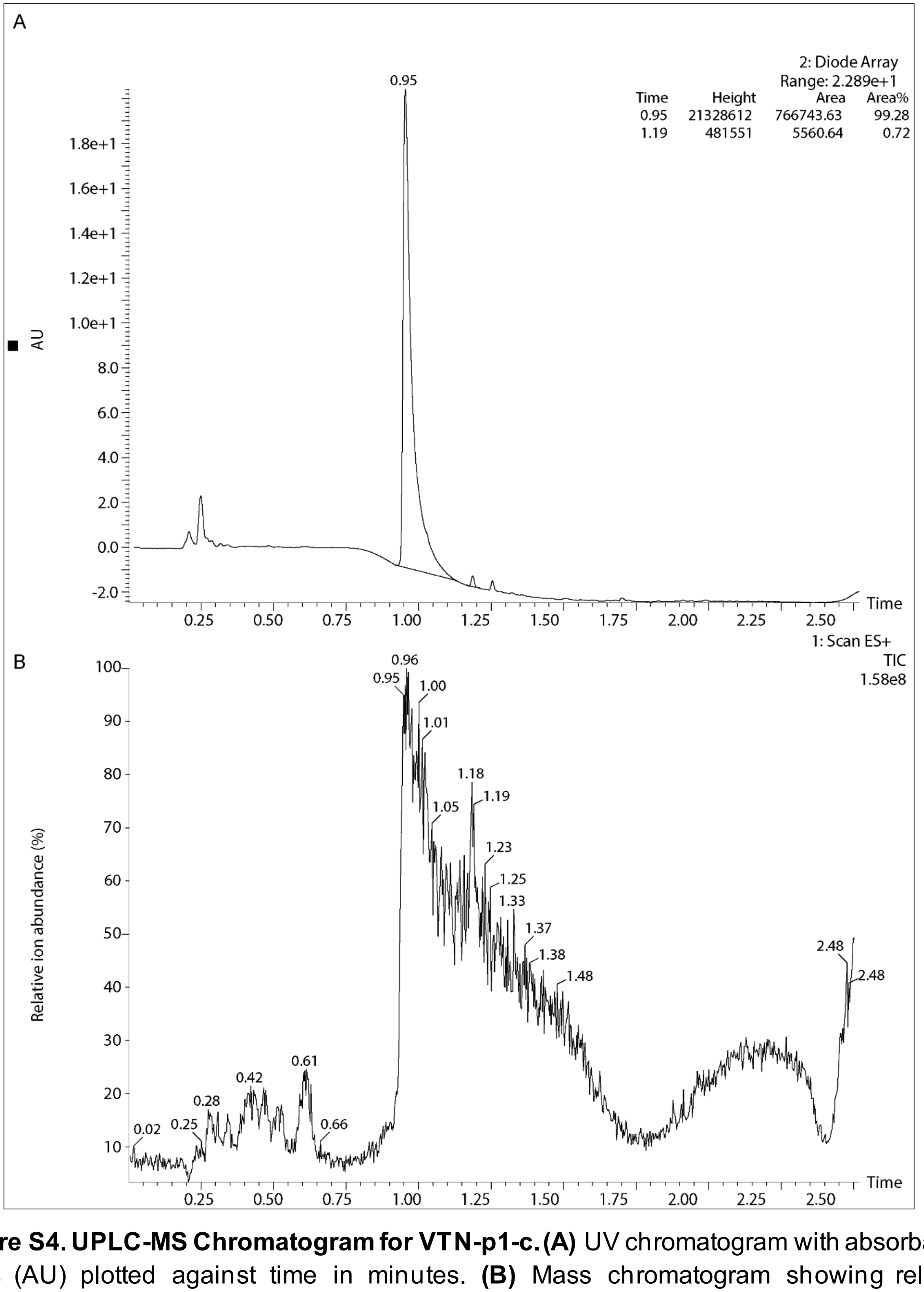

Figure S4. UPLC-MS Chromatogram for VTN-p1-c. (A) UV chromatogram with absorbance units (AU) plotted against time in minutes. **(B)** Mass chromatogram showing relative abundance of ions (%) plotted against time in minutes.

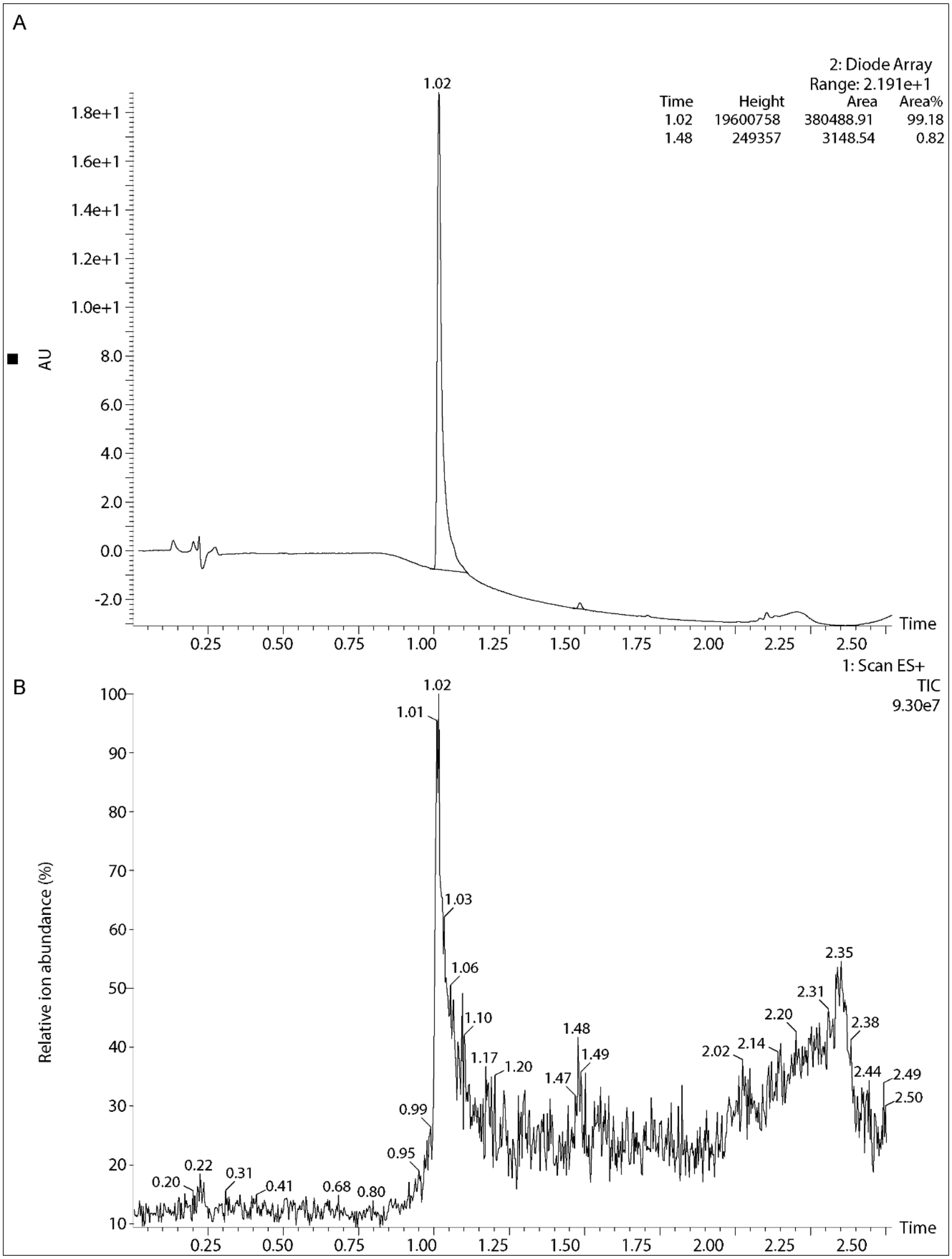

Figure S5. UPLC-MS Chromatogram for THB1-p1-c. Related to Figure 4. **(A)** UV chromatogram with absorbance units (AU) plotted against time in minutes. **(B)** Mass chromatogram showing relative abundance of ions (%) plotted against time in minutes.

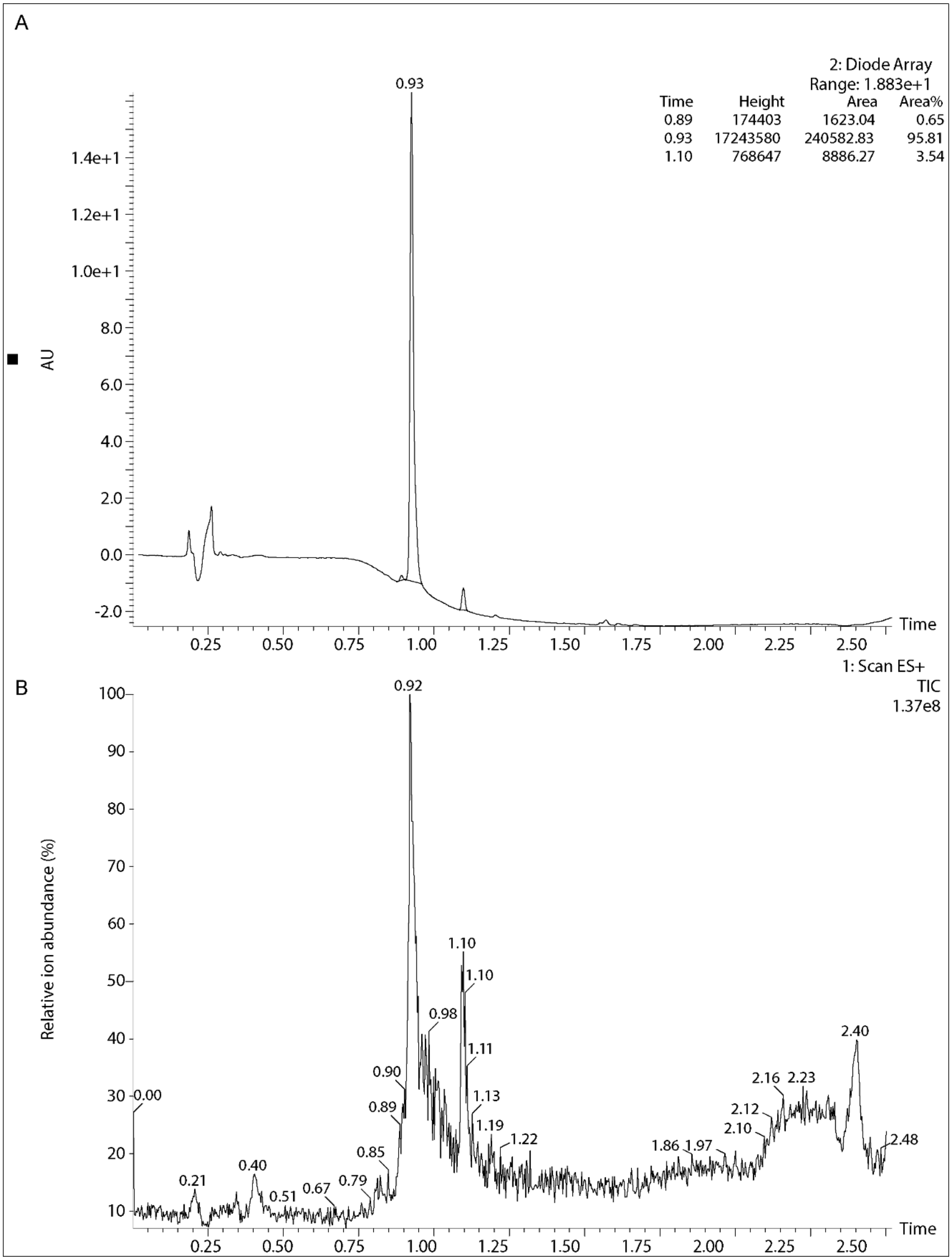

Figure S6. UPLC-MS Chromatogram for FN1-p6-c. (A) UV chromatogram with absorbance units (AU) plotted against time in minutes. **(B)** Mass chromatogram showing relative abundance of ions (%) plotted against time in minutes.

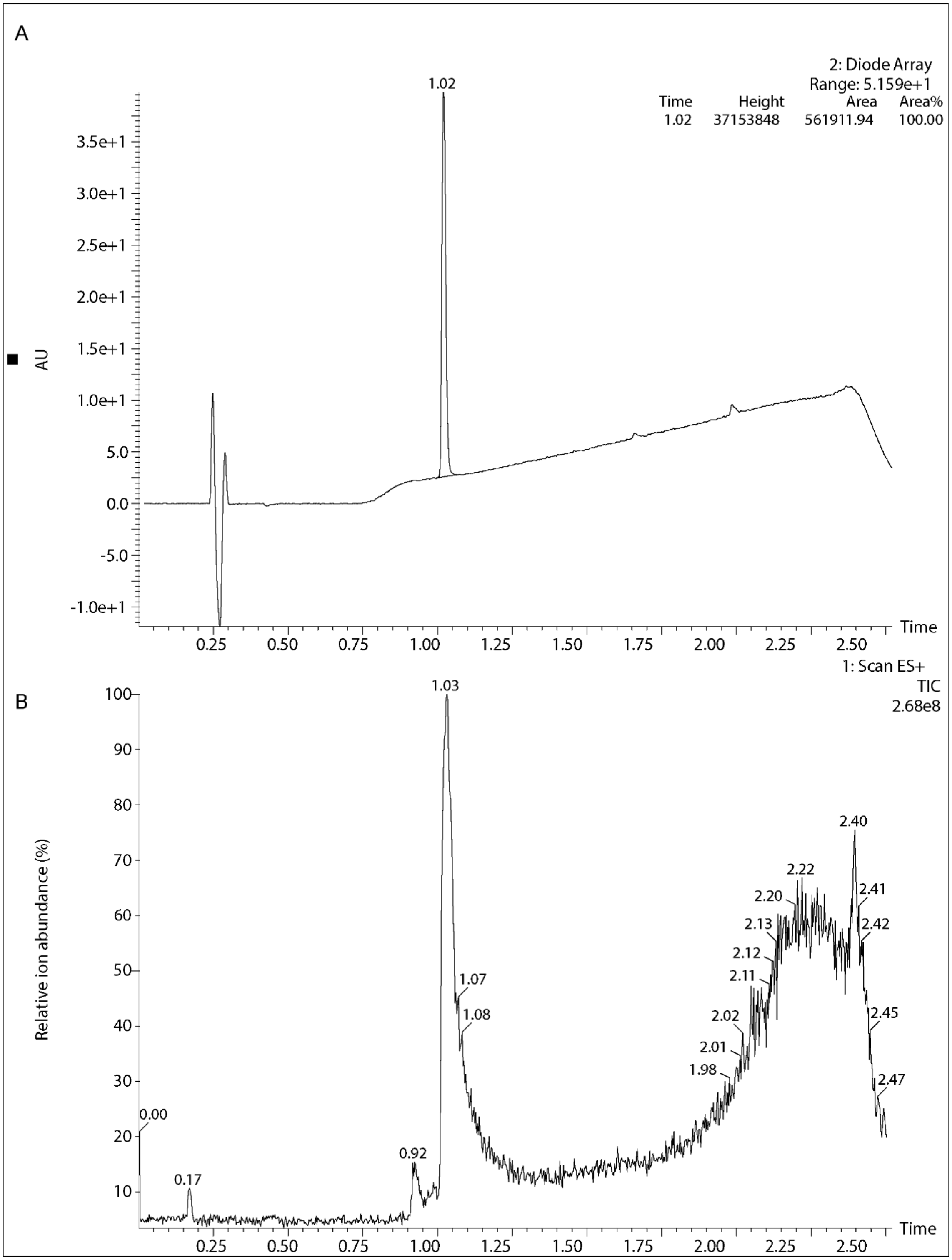

Figure S7. UPLC-MS Chromatogram for LAMα1-p1-b. (A) UV chromatogram with absorbance units (AU) plotted against time in minutes. **(B)** Mass chromatogram showing relative abundance of ions (%) plotted against time in minutes.

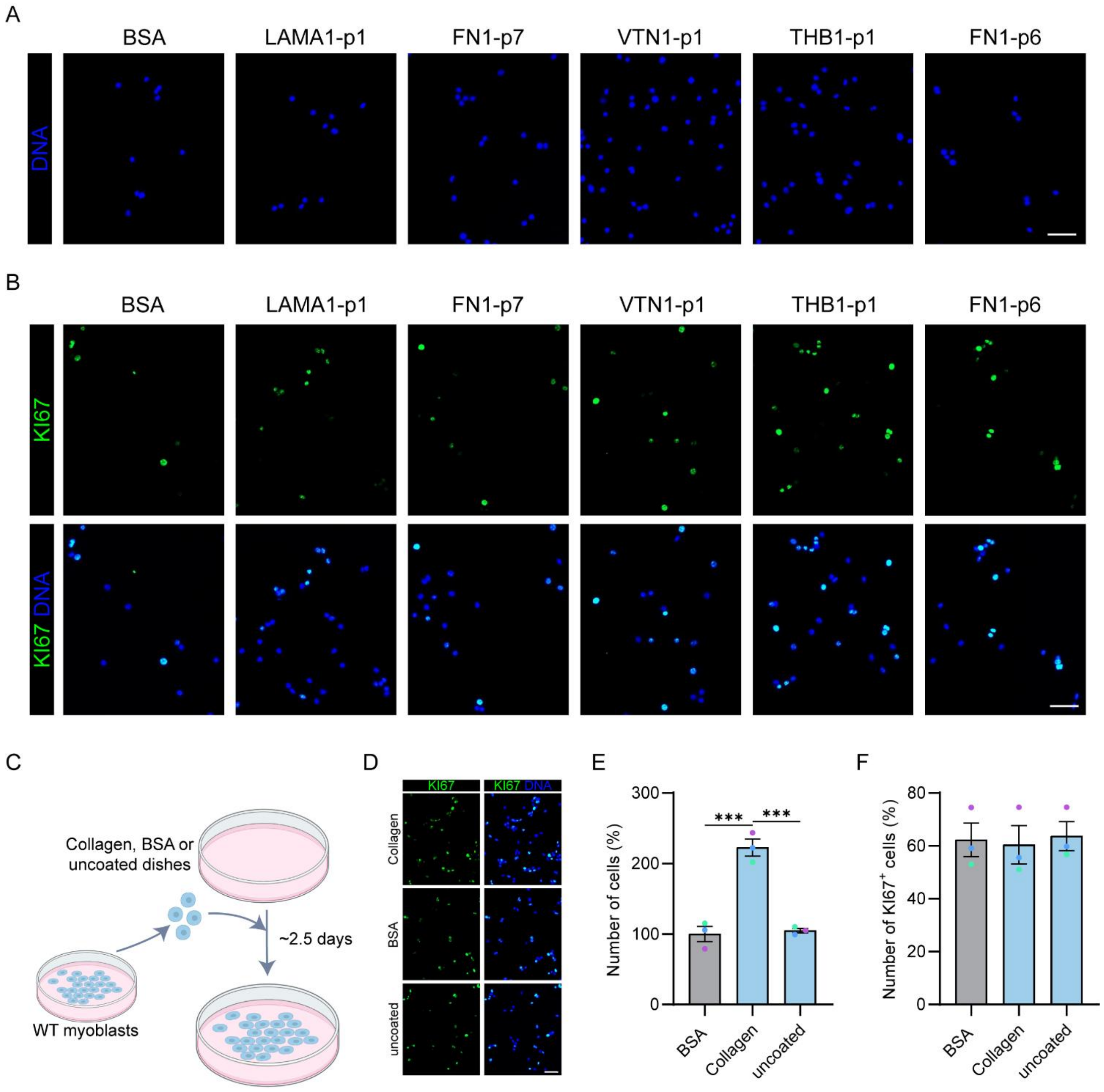

A
BSA
LAMA1-p1
FN1-p7
VTN1-p1
THB1-p1
FN1-p6
DNA
B
BSA
LAMA1-p1
FN1-p7
VTN1-p1
THB1-p1
FN1-p6
KI67
KI67 DNA
C
Collagen, BSA or uncoated dishes
~2.5 days
WT myoblasts
D
KI67
KI67 DNA
Collagen
BSA
uncoated
E
Number of cells (%)
300
200
100
0
***
***
BSA
Collagen
uncoated
F
Number of KI67^{+} cells (%)
80
60
40
20
0
BSA
Collagen
uncoated

Figure S8. Characterization of wildtype myoblast on different substrates. (A) Representative images of wildtype (WT) myoblasts cultured on different substrates corresponding to Figure 4D showing Hoechst positive nuclei in blue (DNA). **(B)** Representative images of WT myoblasts cultured on different substrates corresponding to Figure 4F and 4G showing KI67+ cells in green and Hoechst positive nuclei in blue. **(C)** Schematic workflow for assessing WT myoblast numbers and proliferation after ~2.5 days of culture on Collagen, BSA, and uncoated polystyrene dishes similar to Figure 4E-G. **(D-F)** Representative images and quantification of WT myoblast numbers and the percentage of KI67 positive (KI67+, green) cells among Hoechst positive nuclei after ~2.5 days of culture on BSA, Collagen, or uncoated polystyrene dishes. Data represent means ± sem from n = 3 biological replicates, each corresponding to an independent myoblast line isolated from a separate mouse. Scale bars = 50 µm. P values were calculated using one-way ANOVA with Tukey correction. *P<0.05, **P<0.01, ***P<0.001, ****P<0.0001.

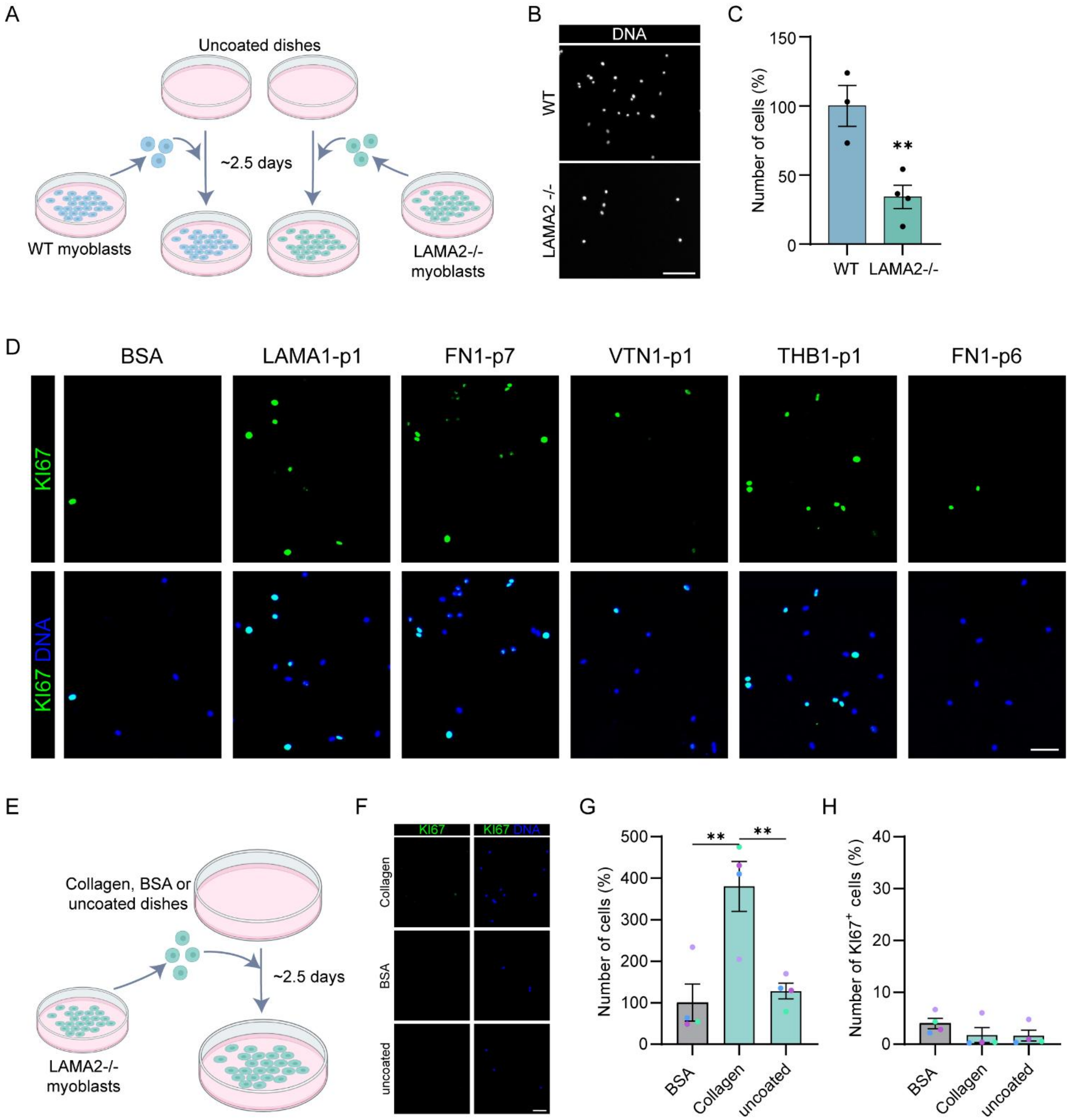
A
Uncoated dishes
~2.5 days
WT myoblasts
LAMA2-/- myoblasts
B
DNA
WT
LAMA2 -/-
C
Number of cells (%)
150
100
50
0
**
WT
LAMA2-/-
D
BSA
LAMA1-p1
FN1-p7
VTN1-p1
THB1-p1
FN1-p6
KI67
KI67 DNA
E
Collagen, BSA or uncoated dishes
~2.5 days
LAMA2-/- myoblasts
F
KI67
KI67 DNA
Collagen
BSA
uncoated
G
Number of cells (%)
500
400
300
200
100
0
**
**
BSA
Collagen
uncoated
H
Number of KI67+ cells (%)
40
30
20
10
0
BSA
Collagen
uncoated

Figure S9. Characterization of laminin-α2-deficient myoblasts on different substrates. (A) Scheme of the experimental design for comparing numbers of WT and laminin-α2-deficient (LAMA2-/-) myoblasts from dystrophic dyW mice ~2.5 days after seeding on uncoated polystyrene dishes. **(B,C)** Representative images and quantification of the number of cells in the experiment outlined in (A). Hoechst (white) was used to stain nuclei (DNA). **(D)** Representative images of LAMA2-/- myoblasts cultured on different substrates corresponding to Figure 4I and 4J showing KI67+ cells in green and Hoechst positive nuclei in blue (DNA). **(E)** Schematic workflow for assessing LAMA2-/- myoblast numbers and proliferation after ~2.5 days of culture on Collagen, BSA and uncoated polystyrene dishes similar to Figure 4H-J. **(F-H)** Representative images and quantification of LAMA2-/- myoblast numbers and the percentage of KI67 positive (KI67+, green) cells among Hoechst positive nuclei (blue) after ~2.5 days of culture on BSA, Collagen, or uncoated polystyrene dishes. Data represent means ± sem from n = 3 (WT) and n = 4 (LAMA2-/-) biological replicates, each corresponding to an independent myoblast line isolated from a separate mouse. Scale bars = 50 µm. P values were calculated using one-way ANOVA with Tukey correction (G,H) or unpaired, two-tailed students *t*-test (C). *P<0.05, **P<0.01, ***P<0.001, ****P<0.0001.

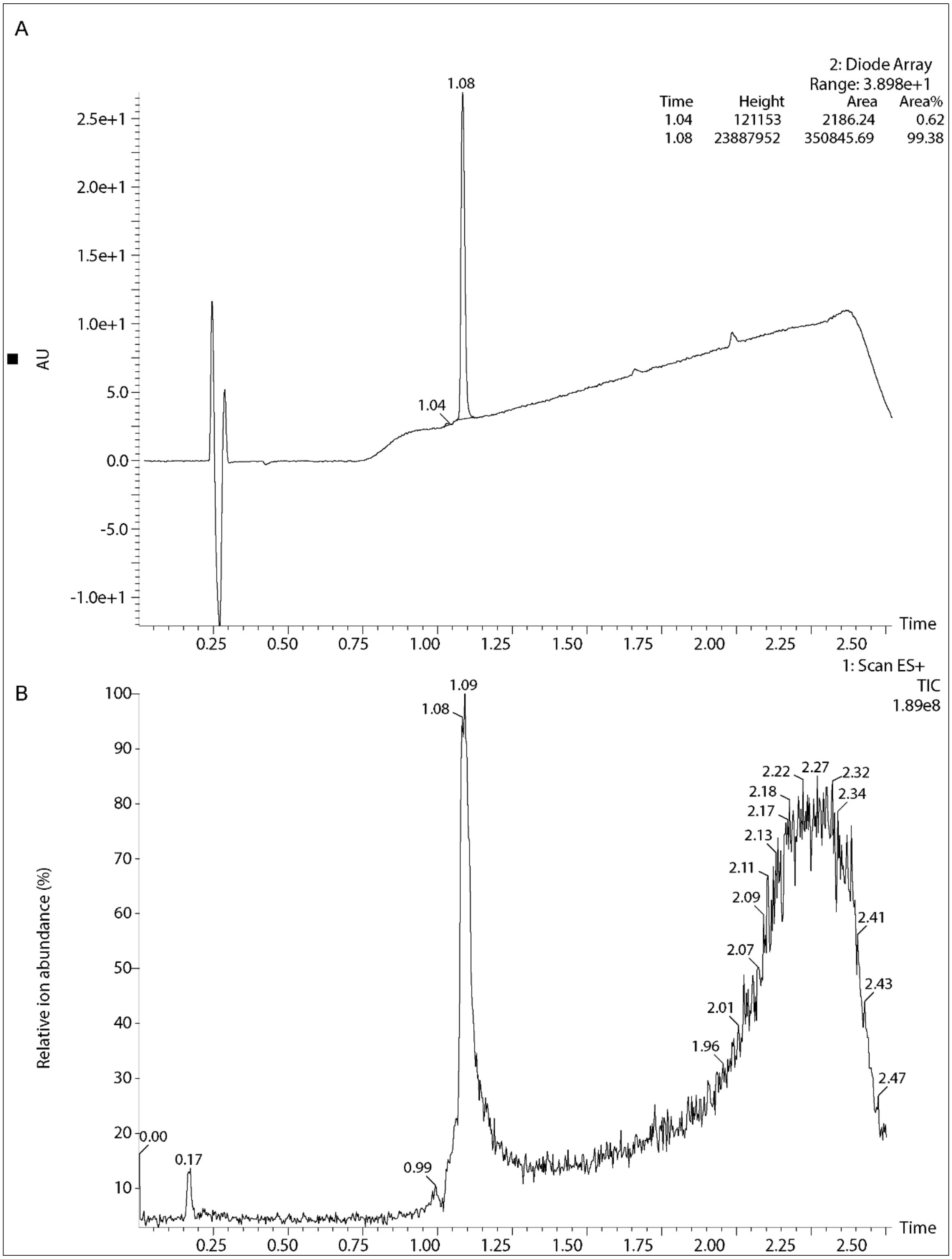

Figure S10. UPLC-MS Chromatogram for LAMα1-p1-mal. (A) UV chromatogram with absorbance units (AU) plotted against time in minutes. **(B)** Mass chromatogram showing relative abundance of ions (%) plotted against time in minutes.

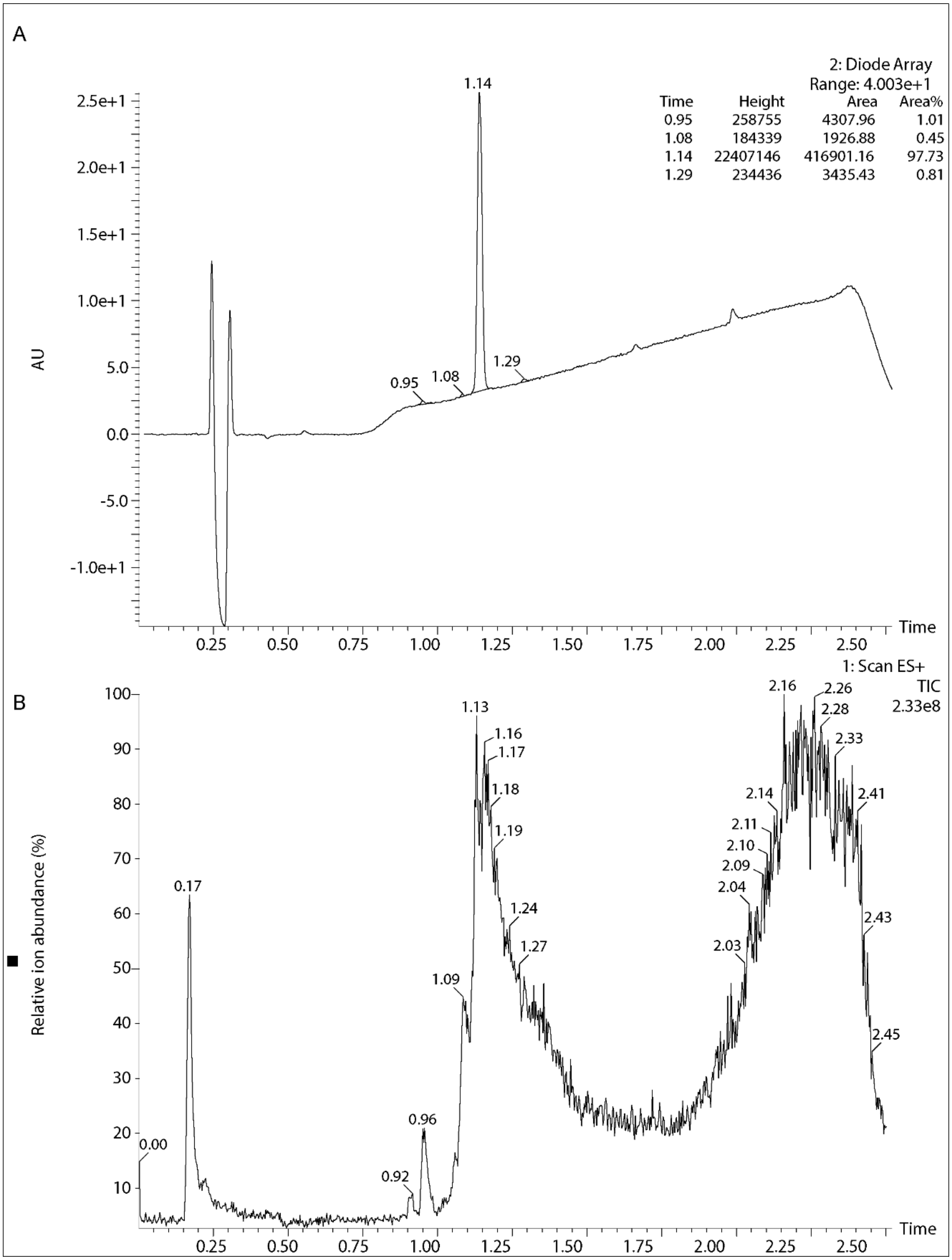

Figure S11. UPLC-MS Chromatogram for LAMα1-p3-b. (A) UV chromatogram with absorbance units (AU) plotted against time in minutes. **(B)** Mass chromatogram showing relative abundance of ions (%) plotted against time in minutes.

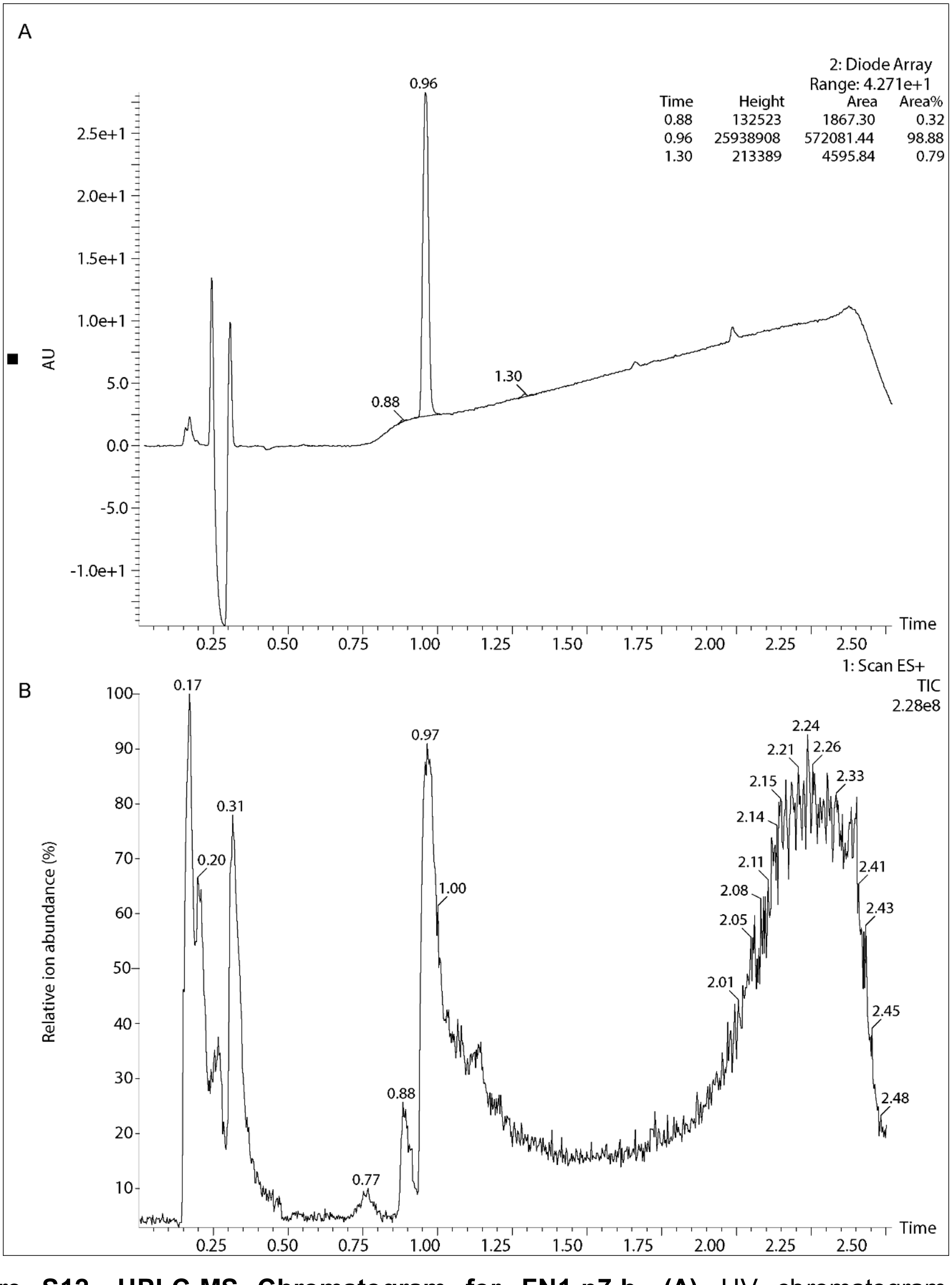

Figure S12. UPLC-MS Chromatogram for FN1-p7-b. (A) UV chromatogram with absorbance units (AU) plotted against time in minutes. **(B)** Mass chromatogram showing relative abundance of ions (%) plotted against time in minutes.

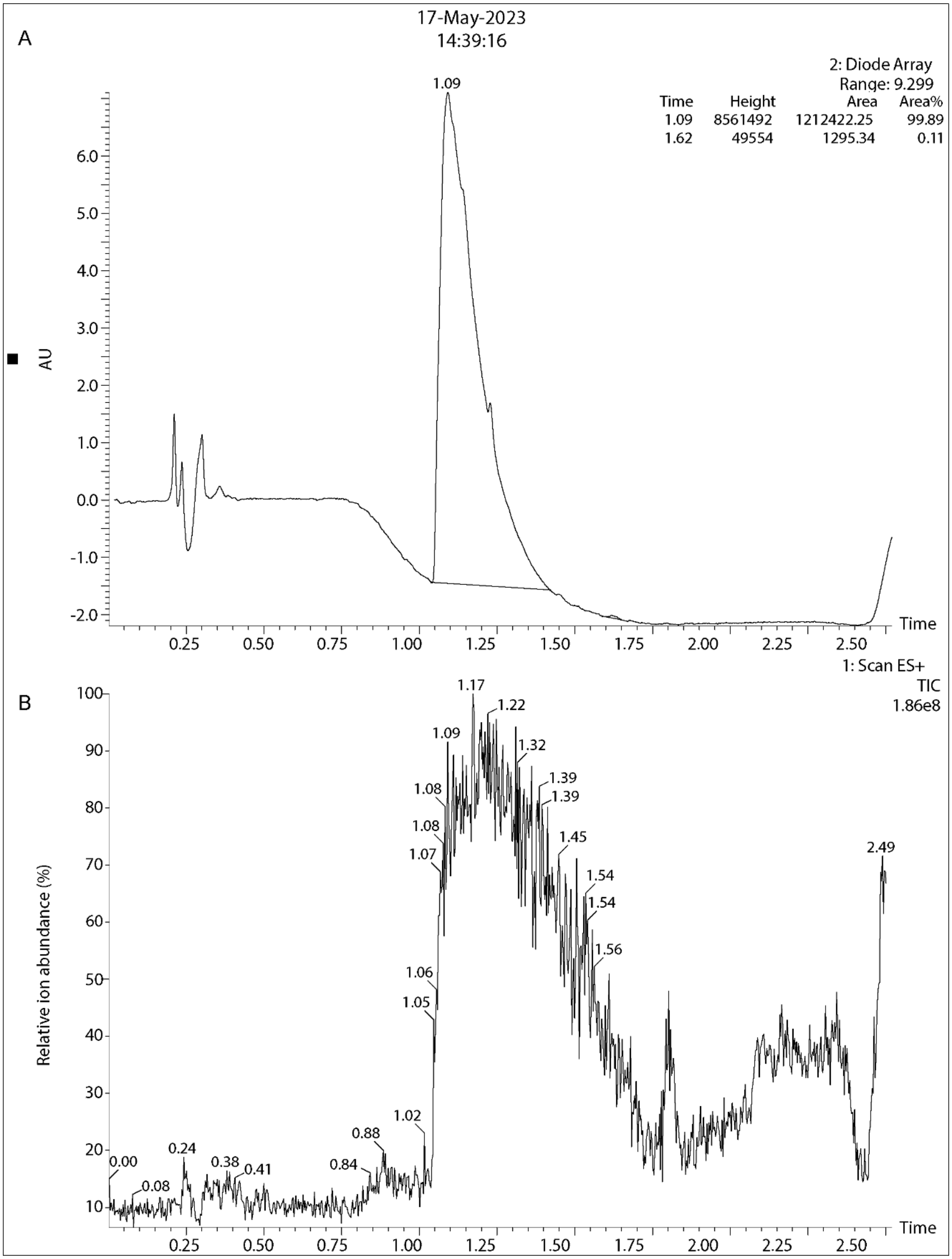

Figure S13. UPLC-MS Chromatogram for VTN-p1-b. (A) UV chromatogram with absorbance units (AU) plotted against time in minutes. **(B)** Mass chromatogram showing relative abundance of ions (%) plotted against time in minutes.

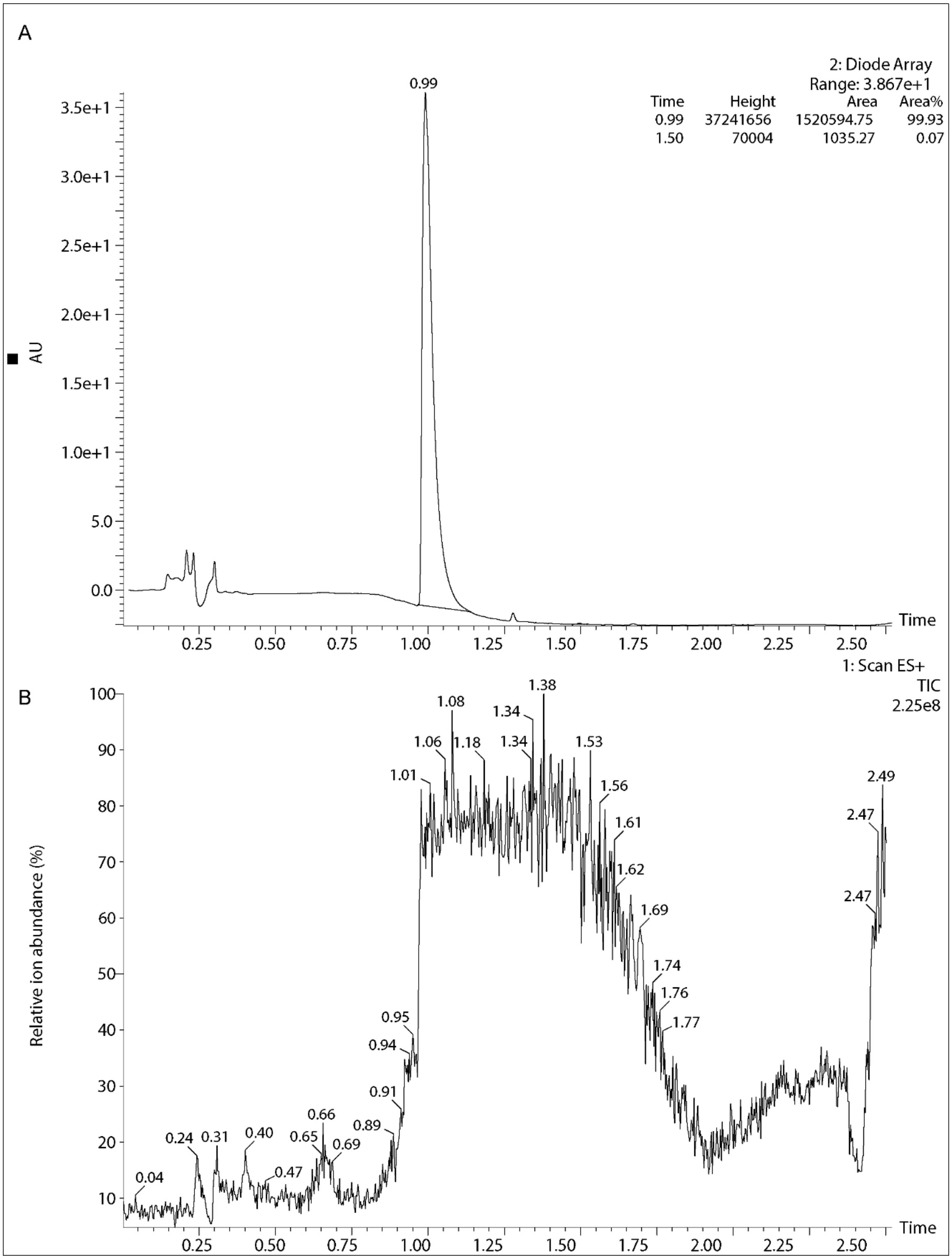

Figure S14. UPLC-MS Chromatogram for THB1-p1-b. (A) UV chromatogram with absorbance units (AU) plotted against time in minutes. **(B)** Mass chromatogram showing relative abundance of ions (%) plotted against time in minutes.

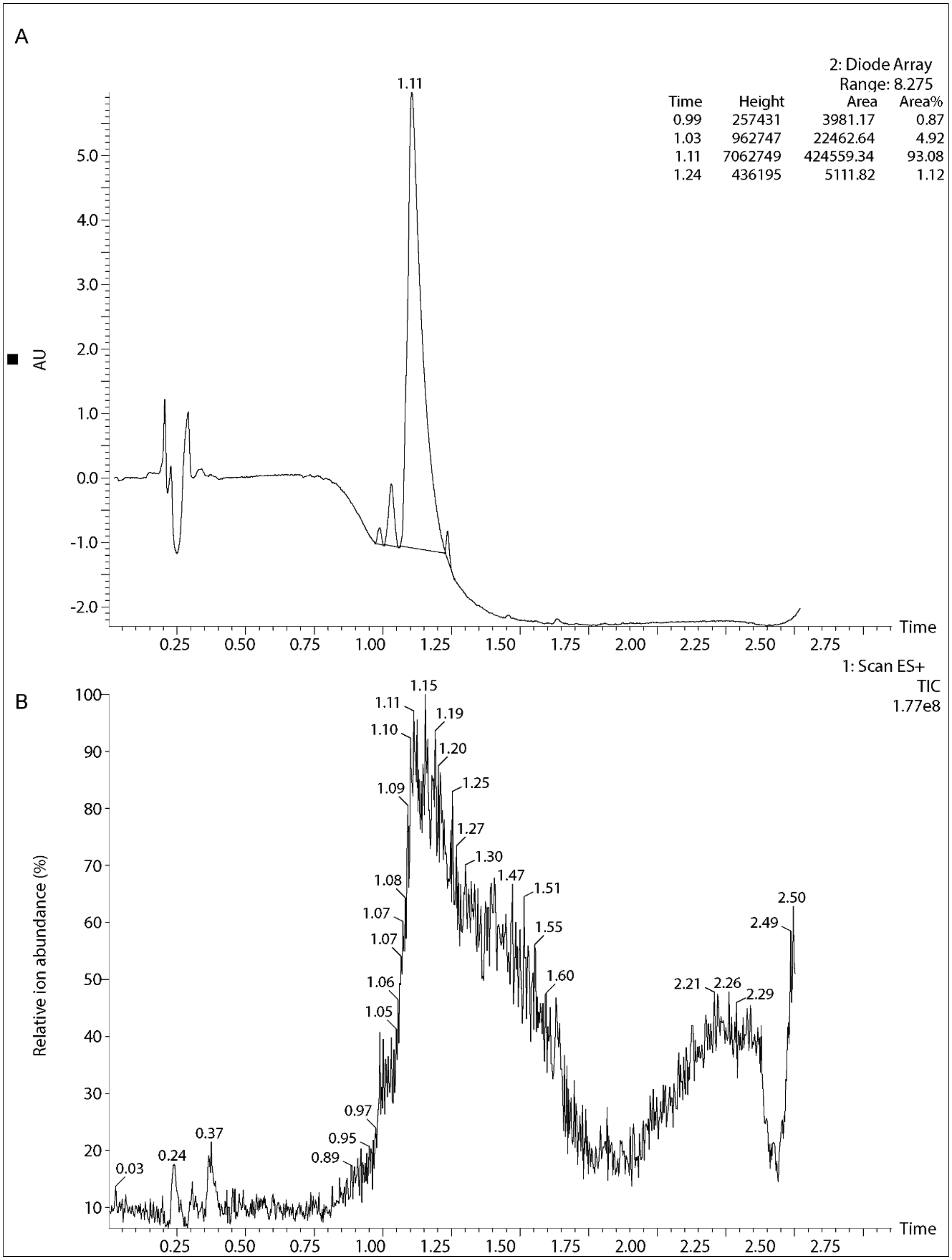

Figure S15. UPLC-MS Chromatogram for FN1-p6-b. (A) UV chromatogram with absorbance units (AU) plotted against time in minutes. **(B)** Mass chromatogram showing relative abundance of ions (%) plotted against time in minutes.

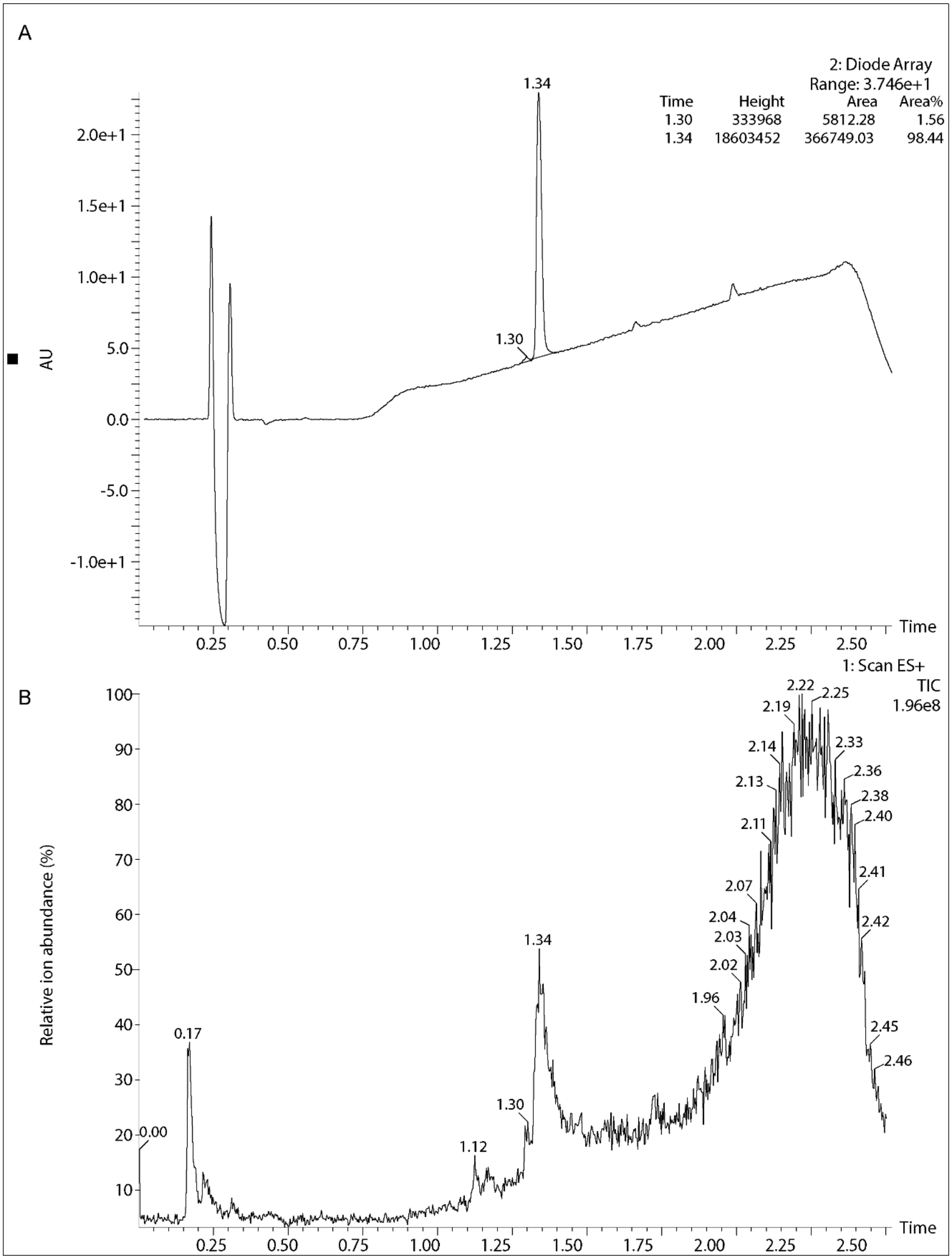

Figure S16. UPLC-MS Chromatogram for SCR-p5-b. (A) UV chromatogram with absorbance units (AU) plotted against time in minutes. **(B)** Mass chromatogram showing relative abundance of ions (%) plotted against time in minutes.
